# Identification and validation of ferroptosis-related genes and immune cell infiltration in thyroid associated ophthalmopathy

**DOI:** 10.3389/fgene.2023.1118391

**Published:** 2023-03-20

**Authors:** Sainan Chen, Jiale Diao, Zifan Yue, Ruili Wei

**Affiliations:** Department of Ophthalmology, Changzheng Hospital of Naval Medicine University, Shanghai, China

**Keywords:** thyroid associated ophthalmopathy, ferroptosis-related gene, immune cell infiltration, lncRNA, GEO

## Abstract

Thyroid associated ophthalmopathy (TAO) is an orbital autoimmune inflammatory disease that is commonly associated with thyroid dysfunction. Although the etiology of TAO is unclear, ROS accumulation and oxidative stress have been closely linked to the pathogenesis of TAO. Ferroptosis is an iron-dependent programmed cell death characterized by intracellular labile iron levels, excessive accumulation of reactive oxygen species (ROS) and lipid peroxidation. Currently, there are few reports regarding the role of ferroptosis in TAO. This article aimed to identify ferroptosis-related genes (FRGs) with diagnostic and therapeutic potential in TAO and explore their relationship with immune cells and lncRNAs. GSE58331 was downloaded from Gene Expression Omnibus (GEO) database. A total of 162 DEGs were identified between 27 TAO samples and 22 health samples from GSE58331, among which six FRGs (*CYBB, CTSB, SLC38A1, TLR4, PEX3*, and *ABCC1*) were obtained. The AUC of *SLC38A1, TLR4, PEX3* in lacrimal gland tissues was greater than 80 which suggested high diagnostic value in TAO. The result of immune cell infiltrate analysis indicated increased infiltration of monocytes (*p* < 0.001), macrophages M0(*p* = 0.039), mast cells activated (*p* = 0.008), and neutrophils (*p* = 0.045) in orbital tissues from TAO patients. Meanwhile, mast cells resting (*p* = 0.043) and macrophages M2 (*p* = 0.02) showed reduced infiltration in TAO samples. There were no gender differences in immune cell infiltration in the TAO patients. Two differentially expressed lncRNAs, *LINC01140* and *ZFHX4-AS1*, in TAO groups were identified as ferroptosis-related lncRNAs. *CYBB-LINC01140-TLR4, CYBB- LINC01140- SLC38A1, TLR4- LINC01140- SLC38A1,* and *CTSB- ZFHX4-AS1- CYBB* may be potential RNA regulatory pathways in TAO. Targeted drugs and transcription factors for differential expressed FRGs were also screened out in our study. *In vitro,* experiments revealed that *CTSB, P*EX3, ABC*C1* and *ZFHX4-AS1(lncRNA)* were differentially expressed in orbital fibroblasts (OFs) between TAO groups and healthy controls at the transcriptional level.

## Introduction

TAO is an orbital autoimmune inflammatory disease that is commonly associated with various thyroid disorders, including Graves’ disease, hypothyroidism, and Hashimoto’s thyroiditis. TAO mainly affects the orbital tissues and may induce several pathological changes such as inflammatory infiltration, retrobulbar fat production, and thickening of the extraocular muscles. Although the clinical features, diagnostic criteria, and treatment strategy of TAO have been reported recently, its ultimate pathogenesis and molecular mechanisms remain unknown. Oxidative stress which indicated the imbalance state of internal oxidative/antioxidant systems plays a crucial role in the process and deterioration of TAO (Bartalena et al., 2003). The target tissue and cells of TAO patients are generally in a state of oxidative stress (Hondur et al., 2008; Choi et al., 2018). Antioxidants, such as selenium and Vitamin C, are recommended by guidelines for patients with mild TAO during the active phase (Bartalena et al., 2021). Ferroptosis, a type of programmed cell death, is closely related to the accumulation of intracellular ROS and lipid peroxidation in cell membranes. However, few studies have explored the role of ferroptosis in TAO.

In the active phase of TAO, the most prominent pathological change is the immune inflammatory response in the orbit tissue. OFs are the target cells in TAO. These cells exhibit mesenchymal stem cell properties and can potentially differentiate into adipose tissue or myofibroblasts in response to different stimuli (Bahn, 2010). Cytokines and chemokines secreted by immune cells can facilitate the differentiation of OFs (Antonelli et al., 2020). Several immune cells, including active T-lymphocytes, monocytes, macrophages, and mast cells, infiltrate the orbit tissue of TAO patients. But previous studies have only focused on individual immune cells. Thus, the infiltration profile of all immune cells in orbital tissue from TAO patients has not been characterized.

The non-coding RNA (ncRNA) refers to a category of RNA that does not encode for a protein. In human genomes, only 2%–3% of the transcriptionally active RNAs have the ability to encode for proteins. Most RNAs are ncRNAs (Kapranov et al., 2007). LncRNAs are ncRNAs with a length greater exceeding 200 nucleotides. The function of lncRNAs in TAO is increasingly being recognized in recent years. A study by Lianqun et al. reported that lncRNAs might be involved in the regulation of extracellular matrix remodeling in TAO orbital adipose/connective tissue (Wu et al., 2021). Zifan Yue constructed an lncRNA-miRNA-mRNA network through high throughput sequencing for orbital tissues and found that the network was associated with the pathogenesis of TAO (Yue et al., 2021). In our study, we identify the differential expressed lncRNAs in orbital tissues of TAO and explore their relationship with ferroptosis.

## Materials and methods

### Microarray data collection

**FIGURE 1:**
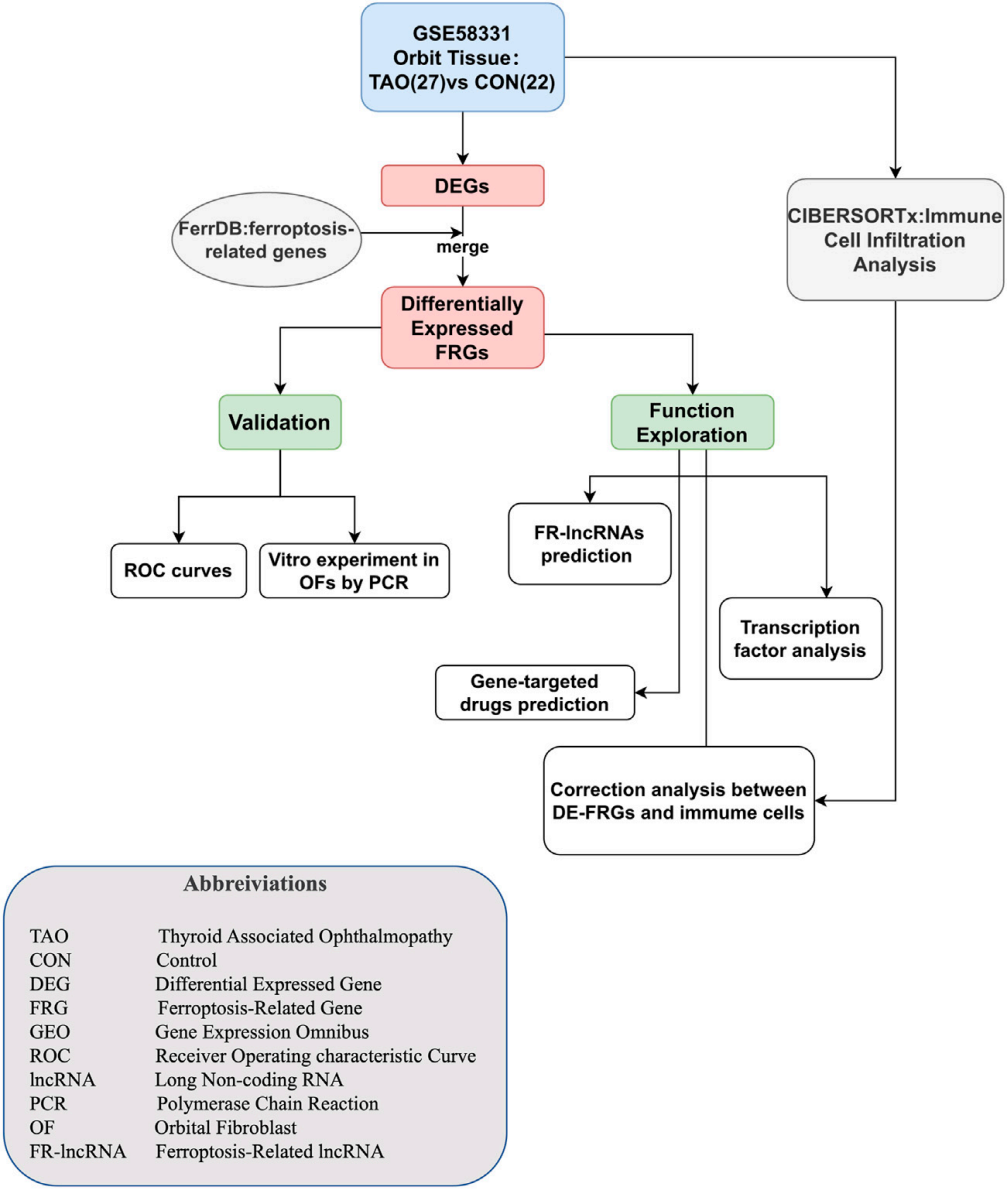
The flow diagram of our study.

GEO (https://www.ncbi.nlm.nih.gov/geo/) is a high-throughput gene expression database that contains microarray, second-generation sequencing, and other high throughput sequencing data. GSE58331, which was established on the platform of GPL570 (Affymetrix Human Genome U133 Plus 2.0 Array) was obtained by searching for thyroid associated ophthalmopathy in GEO. The raw data and annotation files of GSE58331 were acquired from the GEO database. Of the 175 samples in this dataset, 27 and 22 samples of orbital tissue were from TAO patients and healthy people, respectively. These samples were used as the train group to identify DEGs and differential expressed FRGs. Seven TAO and 8 healthy samples of lacrimal gland tissue were selected as the test group to plot ROC curves. We collected the gender information of the samples used in our work and presented in the Supplementary Table S1. The flow chart of the whole research was shown in Figure 1.

### Screening for differential expressed genes (DEGs) and differentially expressed FRGs

ID conversion of raw data was accomplished using Perl based on the annotation files. The Limma package was used to screen out DEGs, setting the selection criterion at |log_2_Fold Change|(log_2_FC)>0.25 and adjusted *p*-value<0.05. FerrDb (http://www.zhounan.org/ferrdb/current/) is the first manually compiled database which is dedicated to ferroptosis regulators and ferroptosis-disease associations. A set of ferroptosis drivers, suppressors, and marker genes were downloaded from FerrDb. To identify differentially expressed FRGs, we intersected genes in the obtained sets with DEGs. A volcano plot and heatmap were employed to visualize the DEGs and differentially expressed FRGs, respectively.

### Establishment of the receiver operating characteristic curve (ROC) curve

The ROC curve was plotted in the R studio using the R package “qROC” to evaluate the diagnosis value of the screened FRGs in TAO. The area under curve (AUC) was calculated to quantitatively assess the power of prediction accuracy. An AUC value more than 0.7 indicated high diagnostic value for the disease.

### Immune cell infiltration analysis

We performed immune cells infiltration analysis using the CIBERSORT algorithm. This algorithm was developed by Newman et al. and is used to estimate the abundance of 22 types of immune cells in gene expression profile data. The source code of CIBERSORT and LM22. txt which includes signature genes and their expression profile in 22 types of immune cell were downloaded from CIBERSORTx (https://cibersortx.stanford.edu/). We calculated the *p*-value for each sample to estimate their heterogeneity of immune infiltration by using the permutation test inside the CIBERSORTx. One sample with the *p*-value greater than 0.05 indicates that there is no statistical difference in immune cell infiltration and would be excluded in the follow-up analysis. Spearman correlation analysis was applied to analyze the correlation between differentially expressed FRGs and immune cells in TAO samples with the filter of Spearman’s R > 0.4 or < -0.04 and *p*-values <0.05. The analysis was also performed using the R packages (“corplot,” “vioplot,” “ggplot2,” and “glment”).

### Identification of the ferroptosis-related lncRNAs

The human genome annotation file GRCh38 was obtained from Genecode (Frankish et al., 2021) and used to distinguish lncRNAs from mRNAs in the gene matrix. The Limma R package was used to identify differential expressed lncRNAs in TAO groups. The Pearson correlation analysis was utilized to investigate the co-expression of differential expressed lncRNAs and FRGs in the gene matrix. The absolute value of Person’s R > 0.4 and *p*-values <0.05 were considered to indicate a significant co-expression relationship. Differential expressed lncRNAs which showed a significant co-expression relationship with differential expressed FRGs were regarded as ferroptosis-related lncRNAs (FR-lncRNAs).

### Prediction of gene-targeted drugs

The DGIdb (Drug-Gene Interaction database) was utilized to predict drugs targeted by the FRGs identified in our study. The DGIdb is a drug-gene interaction database that provides information on the association of genes with their known or potential drugs and has been updated to version 4.0 (Freshour et al., 2021). More detailed information about these drugs were obtained from the DrugBank database (Wishart et al., 2018).

### Construction of gene-transcription factor regulatory network

TRRUST was used to predict the transcription factors that regulate related FRGs. TRRUST (https://www.grnpedia.org/trrust/) is an artificially annotated database of transcriptional regulatory networks for human and mouse which not only contains the target genes corresponding to transcription factors, but also the regulatory relationships between transcription factors.

### Validation of FRGs and lncRNAs in OFs by RT-qPCR

Orbital tissues were obtained from TAO patients who underwent orbital decompression surgery (*n* = 3) and healthy individuals who received oculoplastic surgery (*n* = 3) in the Shanghai Changzhen Hospital. This program has been reviewed and approved by Committee on Ethics of Biomedicine, Second Military Medical University. Each sample was collected with acquiring informed consent and strictly subjected to 1964 Helsinki declaration. Primary OFs were isolated from orbital tissues as mentioned in the previous study (Diao et al., 2020). Briefly, the orbital tissue obtained during surgery was separated into small pieces and neatly placed in a Petri dish containing small amount of DMEM (10% FBS, L-glutamine, 110 mg/L sodium pyruvate, 1% penicillin/streptomycin). Long spindle-shaped fibroblasts could be observed migrating out of the tissue mass after approximately 7 days. Passage and phenotypic validation were performed when the primary fibroblasts grew to confluence. The human OFs at 3-8 passage was selected for the experiment. Total RNA of OFs was extracted using the RNA-Quick Purification Kit (RN001, Yishan, Shanghai, China) and reverse transcribed to cDNA by HiScript^®^ Q RT SuperMix for qPCR (R122-01, Vazyme, Nanjing, Jiangsu, China). The primer sequence of six FRGs, two lncRNAs and GAPDH used as the housekeeping gene are presented in the Supplementary Table S2. After amplification of cDNA by ChamQ Universal SYBR qPCR Master Mix (Q711-02, Vazyme, Nanjing, Jiangsu, China), the fluorescence signal during the exponential amplification phase was collected and analyzed. All results were analyzed as three replicates from three independent experiments.

## Results

### Identification of differentially expressed ferroptosis-related genes

**FIGURE 2:**
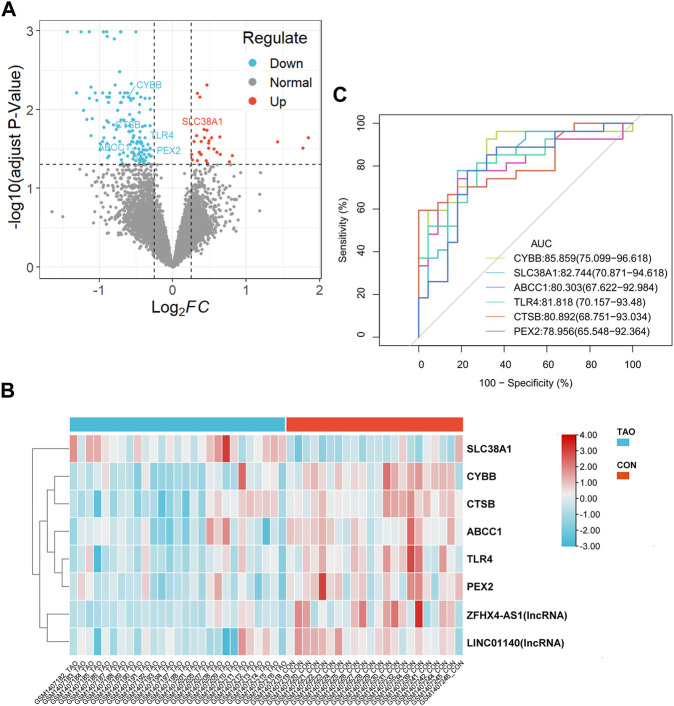
Identification of DEGs and differential expressed FRGs. **(A)** The vocanol plot of DEGs between TAO groups and health groups. The orange plots indicate upregulated genes and the blue plots indicate downregulated genes. **(B)** The heatmap of six differential expressed FRGs and two ferroptosis-related lncRNAs. The blue rectangles indicate low expression and the orange rectangle indicate high expression. **(C)** The ROC curve of six FRGs in the orbital tissue group from patients with TAO and the healthy controls in GSE58331.

A total of 162 DEGs (32 upregulated and 130 downregulated) were identified based on the criteria of log_2_|FC|>0.25 and adjust *p*-value<0.05 **(** Figure 2A and Supplementary Table S3). Six differentially expressed ferroptosis driver genes including *CYBB, CTSB, SLC38A1, TLR4, PEX3*, and *ABCC1* were identified through the intersection of DEGs and ferroptosis-related genes (Table 1). The heatmap showing their expression profile in each sample and the data variation between the two groups is shown in Figure 2B. Except for *SLC38A1*, the other five genes were downregulated in the orbit tissue of TAO patients.

**TABLE 1:** Six differentially expressed FRGs.

Gene symbol	Name	log2FC	Adjust *p*-value
CYBB	Cytochrome b-245 beta chain	−0.604620077	0.006965924
CTSB	cathepsin B	−0.585239204	0.016045506
SLC38A1	solute carrier family 38 member 1	0.425939	0.018077633
TLR4	Toll like receptor 4	−0.444385019	0.019231432
PEX2	peroxisomal biogenesis factor 2	−0.379707628	0.03257408
ABCC1	ATP binding cassette subfamily C member 1	−0.450550612	0.047195025

Annotation: FC, fold change.

### ROC curve of six FRGs in the lacrimal gland samples

The ROC curve of six FRGs based on GSE58331 is presented in Figure 2C.

**FIGURE 3:**
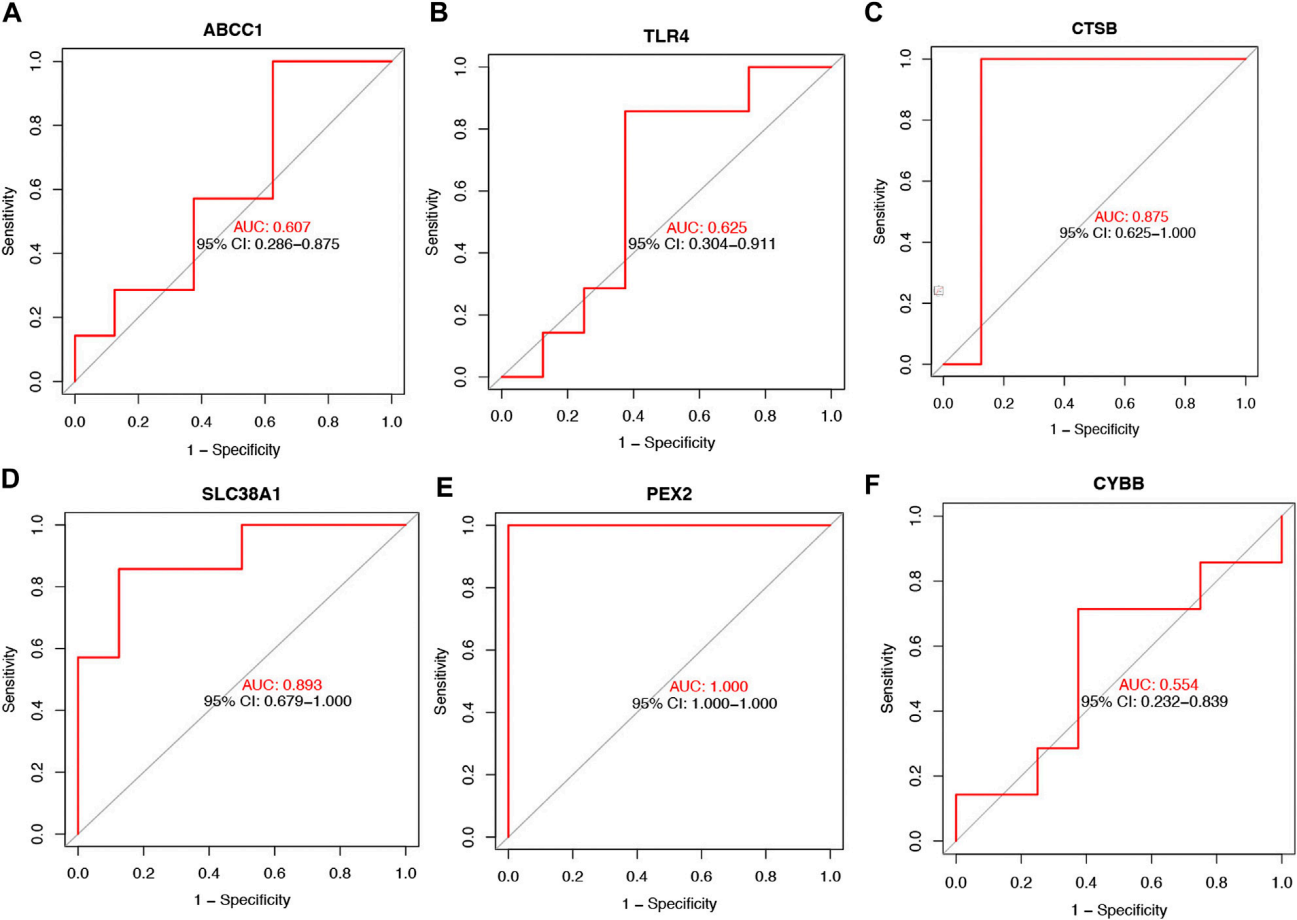
The ROC curve of six FRGs in the lacrimal gland tissue from TAO patients and the health **(A–F)**.

To further determinate the diagnose power of these genes in disease, we plotted the ROC curve in both 49 orbit tissue samples (27 TAO and 22 healthy) and another 15 lacrimal gland samples (7 TAO and 8 healthy) from GSE58331. The AUC value of differentially expressed FRGs was calculated to perform quantitative analysis. The AUC values for six genes in the orbital tissue group were all higher than 80 (Figure 2C). In lacrimal gland tissues groups, the AUC value of *CTSB* (AUC = 0.875; CI: 0.625–1.000), *SLC38A1* (AUC = 0.893; CI:0.679–1.000), and *PEX2* (AUC = 1.000; CI:1.000–1.000) was greater than 80, which indicated high clinical diagnostic power (Figure 3). Meanwhile, we plotted the ROC of TSHR and IGF1R which is the existing TAO molecular markers in both orbit tissue and lacrimal gland based on GSE58331 and compared their AUC value with FRGs in this study. The AUC value of TSHR and IGF1R were not greater than six FRGs in tissues at transcriptional levels (Supplementary Figure S1).

### Immune cell infiltration analysis

**FIGURE 4:**
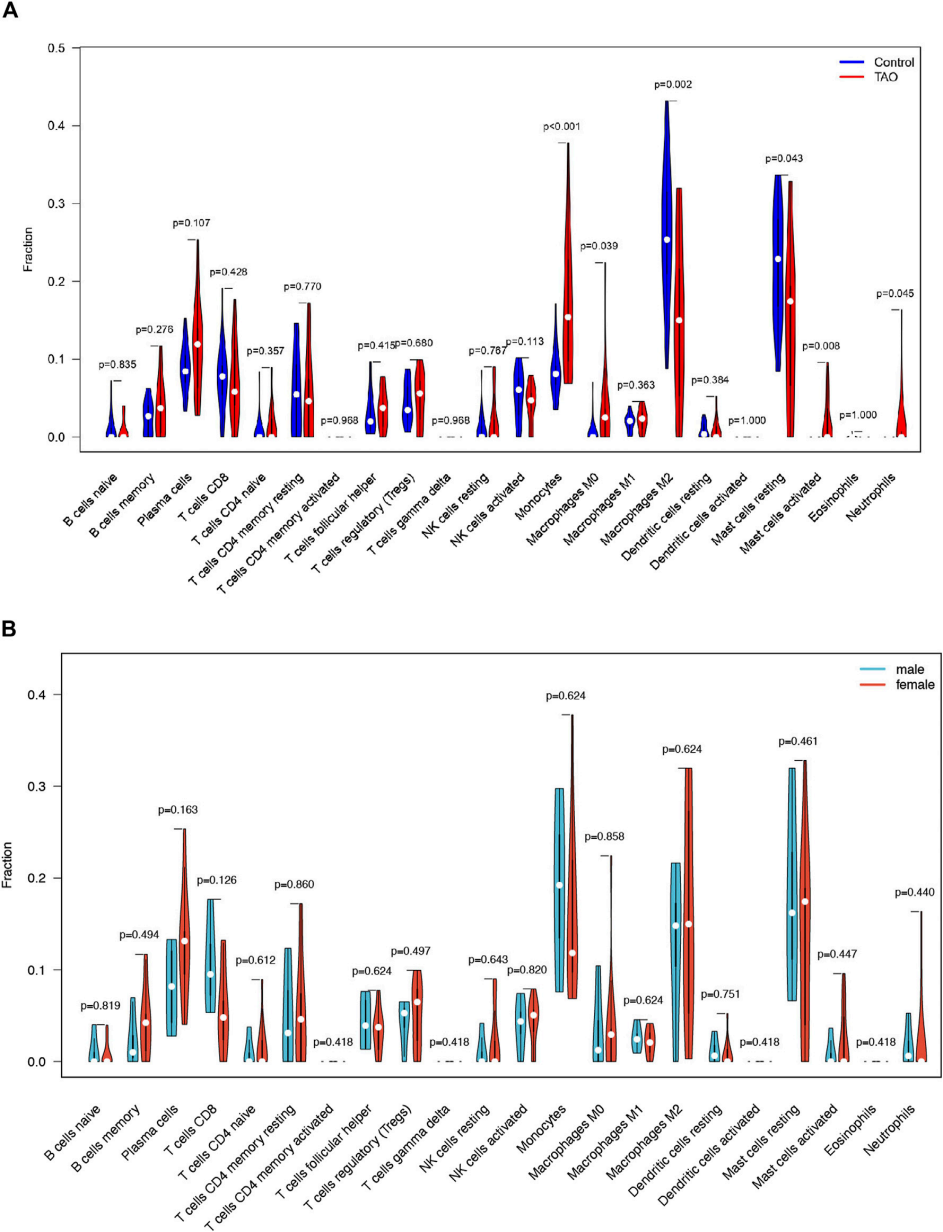
The vioplot of 22 immune cells infiltration. The X coordinate is the name for 22 immune cells and the Y coordinate is the fraction. **(A)** The Orange and blue violin represent expression of immune cells in TAO groups and control groups, respectively. **(B)** The Orange and blue violin represent expression of immune cells in female and male patients with TAO, respectively.

**FIGURE 5:**
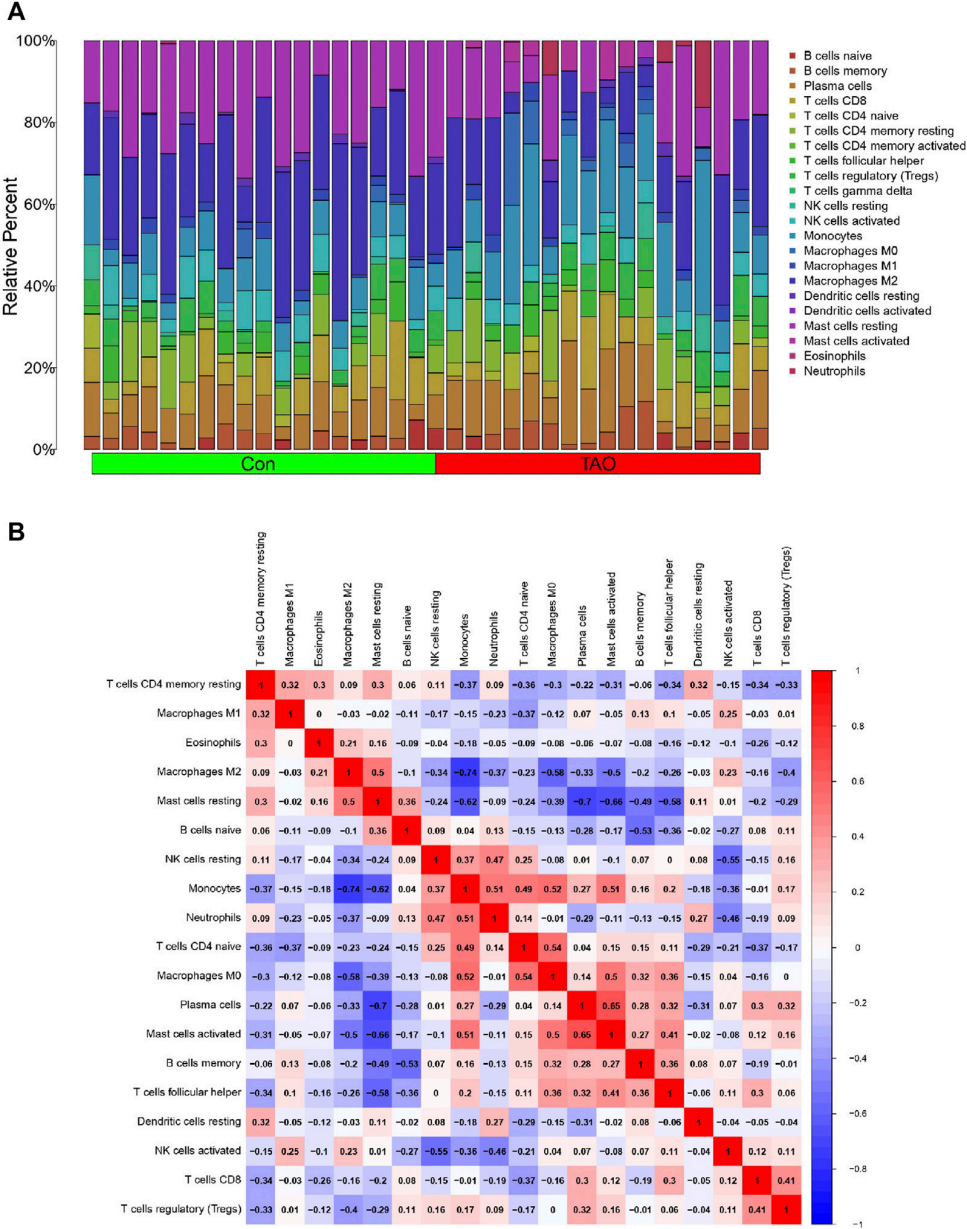
Immune cells infiltration analysis. **(A)** The bar plot of 22 types of immune cell infiltration status in each sample. The green and red rectangle in the bottom represents the control and TAO groups, respectively. The Y coordinate indicate the relative percent of each immune cells in all immune cells. **(B)** Heatmap of immune cells correlation. Both the X and Y coordinate are the names of immune cells. The values inside squares represent the correlation between immune cells, with orange representing positive correlation and blue representing negative correlation.

The violin plot (Figure 4A) of immune cell infiltration indicated high infiltration level of monocytes (*p* < 0.001), macrophages M0(*p* = 0.039), mast cells activated (*p* = 0.008), and neutrophils (*p* = 0.045) in TAO samples. Meanwhile, mast cells resting (*p* = 0.043) and macrophages M2 (*p* = 0.02) which can suppress T cell-mediated immune responses exhibited low infiltration level in TAO samples (Table 2). No gender differences regarding the immune microenvironment were observed in the TAO samples (Figure 4B). The bar plot (Figure 5A) revealed the relative percentage of 22 types of immune cells in each sample. The correlations among 19 types of immune cells (T cells CD4 memory activated, T cells gamma delta, and dendritic cells activated were not included in the analysis because they did not show infiltration in all samples) are presented in the correlation heatmap (Figure 5B). Early inflammatory response in TAO is characterized by monocytes infiltration. In our results, monocytes showed a significant negative correlation with macrophages M0 and mast cells activated but showed a positive correlation with macrophages M0, mast cells activated, neutrophils, and T cells CD4 naïve. Besides, macrophages M2 and monocytes (R = −0.74), mast cells resting and plasma cell (R = −0.7), mast cells resting, and mast cells activated (R = −0.66), T cells follicular helper and mast cells resting (R = −0.58), natural killer (NK) cells activated and NK cells resting (R = −0.55) showed negative relationships. There was a positive relationship between mast cells resting and macrophages M2 (R = 0.5), mast cells activated and plasma cell (R = 0.65), NK cells resting and neutrophils (R = 0.47).

**TABLE 2:** Differential infiltrated Immune Cells.

Immune cell type	*p*-value	Infiltration
Monocytes	0.000603934	increasing
Macrophages M0	0.03906526	increasing
Macrophages M2	0.00217286	decreasing
Mast cells resting	0.04250673	decreasing
Mast cells activated	0.007912656	increasing
Neutrophils	0.045377433	increasing

### Correlation between differentially expressed ferroptosis-related genes and differential infiltrated immune cells in TAO

**FIGURE 6:**
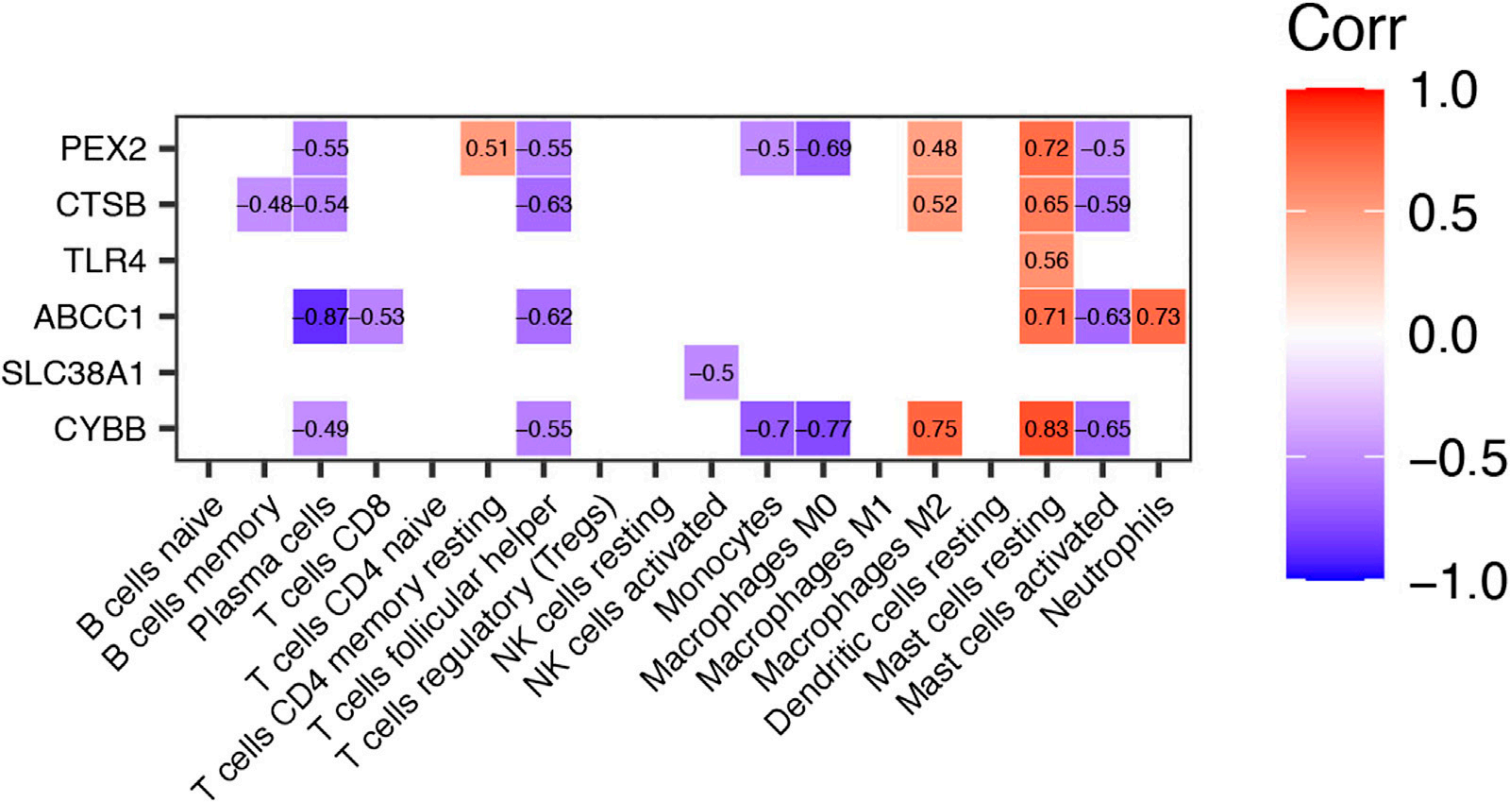
Correlation Heatmap of Differentially expressed FRGs and Immune Cells in TAO groups. The value within each square is the Spearman R and only those values where Spearman R > 0.4 or < −0.4 and *p*-value <0.05 are displayed.

To explore the potential function of differential expressed FRGs (*CYBB, CTSB, SLC38A1, TLR4, PEX2* and *ABCC1*) in the pathogenesis of TAO, we further analyzed the relationship between these genes and 18 immune cells (T cells CD4 memory activated, T cells gamma delta, eosinophils, and dendritic cells activated were not included in the analysis because they did not show infiltration in TAO samples) in TAO groups. The results were presented in a correlation heatmap and were selected based on Spearman’s R > 0.4 and *p*-value<0.05 (Figure 6). Noticeably, *ABCC1* exhibited a significant strong positive correlation with mast cells resting (R = 0.71) and neutrophils (R = 0.73), but a negative correlation with plasma cells (R = −0.87). *CYBB* showed a significant strong positive correlation with macrophages M2 (R = 0.75) and mast cells resting (R = 0.83), but a negative correlation with monocytes (R = −0.7) and macrophages M0 (R = 0.77). A significant strong positive correlation was observed between *PEX2* and mast cells resting (R = 0.72).

### Identification of FR-lncRNAs in TAO

**FIGURE 7:**
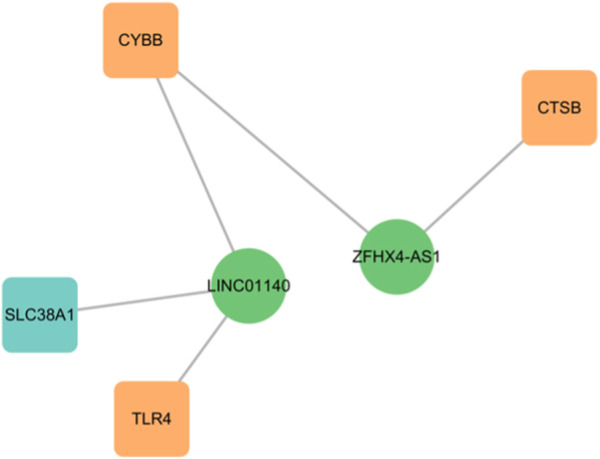
The network of FRGs and FR-lncRNAs.

By applying the criteria of Pearson’s R > 0.4, two lncRNAs (*LINC01140* and *ZFHX4-AS1*) were identified from the DEGs. *LINC01140* exhibited a significant positive correlation with *CYBB* (R = 0.57, *p* < 0.01) and TLR4 (R = 0.46, *p* < 0.05), but a negative correlation with *SLC38A1* (R = −0.64, *p* < 0.001). *ZFHX4-AS1* showed a significant positive correlation with *CTSB* (R = 0.64, *p* < 0.001) and *CYBB* (R = 0.53, *p* < 0.01) (Figure 7; Table 3). *CYBB-LINC01140-TLR4, CYBB- LINC01140- SLC38A1, TLR4- LINC01140- SLC38A1,* and *CTSB- ZFHX4-AS1- CYBB* were considered to be potential RNA regulatory pathways involved in the pathogenesis of TAO.

**TABLE 3:** Correlation of Differentially Expressed FRGs and two lncRNAs.

Gene symbol	lncRNA	Pearson’s R	*p*-value	Regulation
CYBB	LINC01140	0.568424854	0.001978647	postive
SLC38A1	LINC01140	−0.643291319	0.00029516	negative
TLR4	LINC01140	0.464992002	0.01453072	postive
CTSB	ZFHX4-AS1	0.641075308	0.000314508	postive
CYBB	ZFHX4-AS1	0.529765165	0.004483788	postive

Annotation: Pearson’s R, Pearson’s correlation coefficient.

### Prediction of targeted drugs and transcription factors for FRGs

**FIGURE 8:**
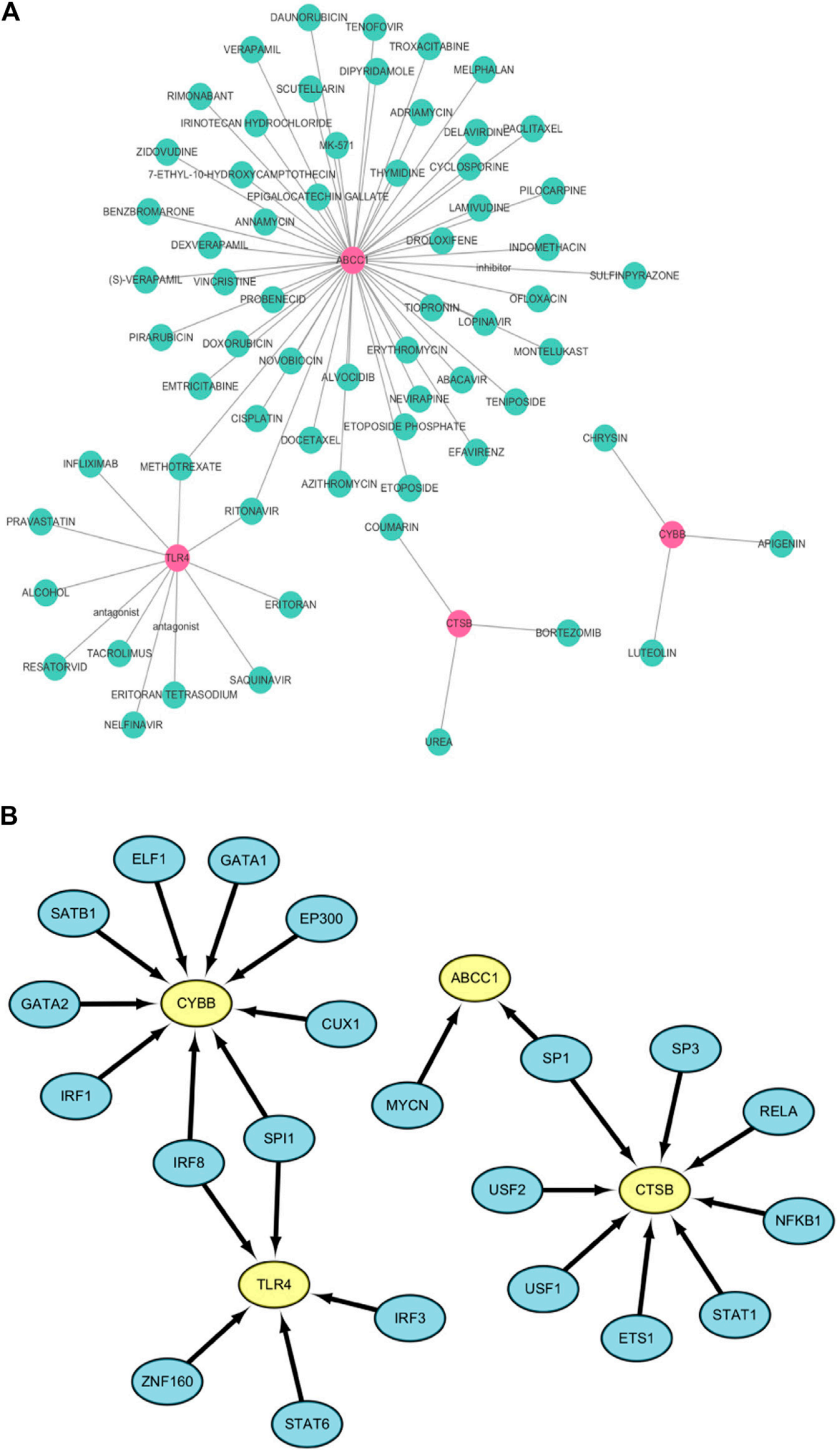
**(A)** The network of FRGs and their target drugs. **(B)** The gene-transcription factor regulatory network.

The DGIdb database was used to predict drugs that interact with the six ferroptosis related genes. Fifty targeted drugs for *ABCC1*, 11 targeted drugs for *TLR4*, 3 targeted drugs for *CTSB* and 3 targeted drugs for *CYBB* were identified in the database. However, drugs that were targeted by *SLC38A1* and *PEX2* were not obtained. These results were visualized by the Cytoscape software as a gene-targeted drugs network shown in Figure 8A. The detailed information, including types, chemical formulas, and structure diagrams of these drugs is presented in the (Supplementary Table S4). Notably, 21 transcription factors for four FRGs (*CYBB, CTSB, TLR4, ABCC1*) were screened out in the TRRUST database. Nine transcription factors for *CYBB*, 8 transcription factors for *CTSB*, 5 transcription factors for *TLR4* and 2 transcription factors for *ABCC1* were also identified (Figure 8B). Among these, *CYBB* and *TLR4* share two common transcription factors (*SPI1* and *IRF8*) whereas *CTSB* and *ABCC1* share one common transcription factor (*SP1*). Transcription factors that regulate *SLC38A1* and *PEX2* were not obtained.

### Validation of FRGs and FR-lncRNAs in OFs by PCR

**FIGURE 9:**
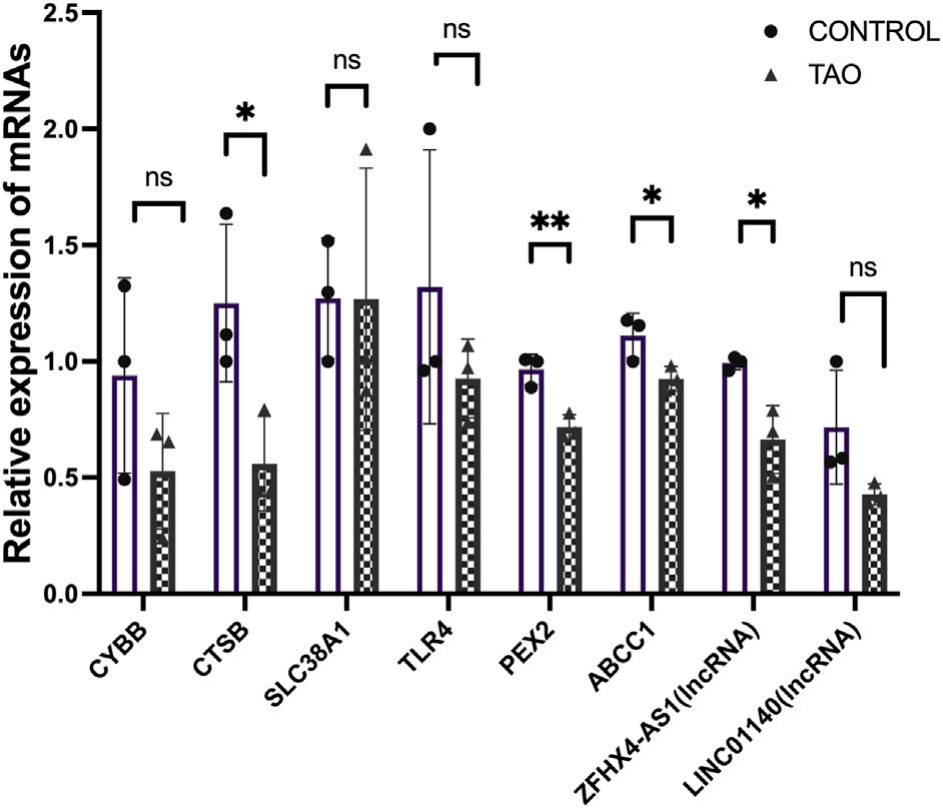
Experimental validation of six FRGs and FR-lncRNAs in OFs cultured from the healthy control and TAO patients. Ns: *p* > 0.05, *p < 0.05, **p < 0.01.

We further validated the expression of the identified differentially expressed FRGs (*CYBB, CTSB, SLC38A1, TLR4, PEX2* and *ABCC1*) and two FR-lncRNAs (*LINC01140* and *ZFHX4-AS1*) in cultured OFs from TAO patients and healthy controls (Figure 9). The results indicated that the expression of *CTSB, PEX2, ABCC1* and *ZFHX4-AS1*were significantly down-regulated in TAO groups which is consistent with our analysis results. Though not significant, the expression of *CYBB* and *TLR4* tended to be decreased in OFs from TAO patients.

## Discussion

In our study, six ferroptosis driver genes (*CYBB, CTSB, SLC38A1, TLR4, PEX2* and *ABCC1*) were identified and all of them exhibited relatively low expression levels in orbital tissues of TAO patients, except for *SLC38A1*. This result implied that ferroptosis might be suppressed in the pathogenesis of TAO. Ma and his colleagues identified ferroptosis resistance in OFs cultured from TAO patients. These cells presented with higher viability than OFs obtained from healthy individuals after cystine deprivation, which is a frequently used method for stimulating the genesis of ferroptosis (Lanzolla et al., 2020). In research on aging diseases, senescence was shown to make cells extremely resistant to ferroptosis due to the accumulation of intracellular iron (Masaldan et al., 2018). Aging was also considered a risk factor involving in the development of TAO (Hiromatsu et al., 2014; Bartalena et al., 2020). Another possible factor contributing to this result might be the status of the thyroid function. The orbital samples of TAO in GSE58331 were collected from patients whose thyroid function had returned to normal.

These FRGs are also meaningful, besides suggesting the status of ferroptosis from TAO. Both radiological and laboratorial evidence suggested the involvement of lacrimal gland in ocular surface damage in TAO patients (Eckstein et al., 2004; Harris et al., 2012; Huang et al., 2014). Thus, we further selected 15 lacrimal gland samples as the test group in GSE58331 to validate the importance of these FRGs in TAO through ROC curve. The AUC value of *CTSB, SLC38A1,* and *PEX2* were greater than 80 in the test group. Meanwhile, PCR results suggested that the expression levels of *CTSB, PEX2* and *ABCC1* were significantly lower in OFs in TAO groups at transcriptional level. This evidence supported our bioinformatics works and confirmed the critical role played by these genes.

Noticeably, PEX2 exhibited the strongest clinical diagnostic power among six FRGs and its expression has also been validated *in vitro* experiments. PEX2 is the essential molecule for peroxisomes to maintain its normal biological functions. It could mediate the autophagy of peroxisomes by ubiquitinate peroxisomal membrane proteins as cells were subjected to amino acid starvation (Sargent et al., 2016). The peroxisome is an organelle that plays a critical role in the metabolism of lipid and ROS (He et al., 2021). Adipogenesis and oxidative stress are the two key pathophysiological changes in TAO (Bahn, 2010). However, the research on the function of peroxisome in TAO has yet been performed so far. The identification and validation of PEX2 in our study might indicated the existence of peroxisome disfunction in TAO.

Two FR-lncRNAs (*LINC01140* and *ZFHX4-AS1*) which were differentially expressed in TAO orbital tissue were identified in this study. *LINC01140* was found to be specifically expressed in lung and breast cancer (Li et al., 2020; Xia et al., 2021) compared to normal tissues. In cellular experiments, *LINC01140* mitigated the inflammatory response of macrophages (He et al., 2020) and modulated macrophage M2 polarization (Wu et al., 2020). *ZFHX4-AS1* is a novel lncRNA which has been primarily investigated in cancer diseases (Li et al., 2019). Both of *LINC01140* and *ZFHX4-AS1* were downregulated in TAO groups and closely linked with the related FRGs.

In the early stages of TAO, inflammatory changes present as infiltration of various immune cells in the orbital tissue. In previous studies, immunohistochemical staining demonstrated a significant increase in the number of T-lymphocytes, B-lymphocytes (Rotondo Dottore et al., 2018), monocytes, macrophages, and mast cells (van Steensel et al., 2012) in orbital tissue of TAO patients. These immune cells can secrete different cytokines and chemokines and regulate the differentiation of OFs. The immune cell infiltration analysis showed significantly increased monocyte, activated mast cells and neutrophil infiltration in patients with TAO, which is consistent with previous studies. Macrophages in previous research exhibited phenotypic heterogeneity and it differentiated according to the inducing factors in concomitance with the acquired phenotypic and functional characteristics, which is called macrophage polarization (Funes et al., 2018). We identified an increased proportion of macrophage M0 in TAO patients. Macrophage M2 mainly serves an anti-inflammatory function. We reported a reduction in macrophage M2 infiltration in orbit tissue obtained from patients with TAO. As TAO is more prevalent in young women, we have further explored the differences in immune cell infiltration between male and female patients. However, probably due to insufficient sample size, we observed no remarkable gender differences.

This work was conducted using the latest and most reasonable algorithms and the most rigorous processes. We identified six differentially expressed FRGs (*CYBB, CTSB, SLC38A1, TLR4, PEX3* and *ABCC1*) in TAO patients. ROC curve and experimental validation demonstrated that the importance of several genes was not limited to the single gene matrix but may have other benefits. Their potential diagnostic power and mechanism are worthy of further investigation.

Nevertheless, the limitation of our study should be noted. First, microarray data used in our work was obtained from the public database. Thus, the characteristic of each subject is limited, like age, duration of TAO, previous treatment, and clinical activity score (CAS). It is difficult to investigate the relationship of gene expression level and clinical feathers. Second, the research of genes in our study was limited to the transcriptional level. In the future, we would continue to focus on the expression of these genes at the protein level and their biological functions in TAO.

## Data Availability

The original contributions presented in the study are included in the article/Supplementary Material, further inquiries can be directed to the corresponding author.

## Abbreviations

AUC, Area Under Curve; CAS, Clinical Activity Score; DEG, Differential Expressed Genes; FRG, Ferroptosis-Related Gene; FR-lncRNA, Ferroptosis-Related lncRNA; GEO, Gene Expression Omnibus; lncRNA, Long Non-coding RNA; NcRNA, Non-coding RNA; OF, Orbital Fibroblast; ROC, Receiver Operating characteristic Curve; RT-qPCR, Real-time Quantitative Polymerase Chain Reaction; TAO, Thyroid Associated Ophthalmopathy.
