# Supplementary figures and images for: Identification and validation of ferroptosis-related genes and immune cell infiltration in thyroid associated ophthalmopathy
Source: Front Genet. 2023 Mar 20;14:1118391. doi: 10.3389/fgene.2023.1118391 (PMC10067720; doi:10.3389/fgene.2023.1118391)

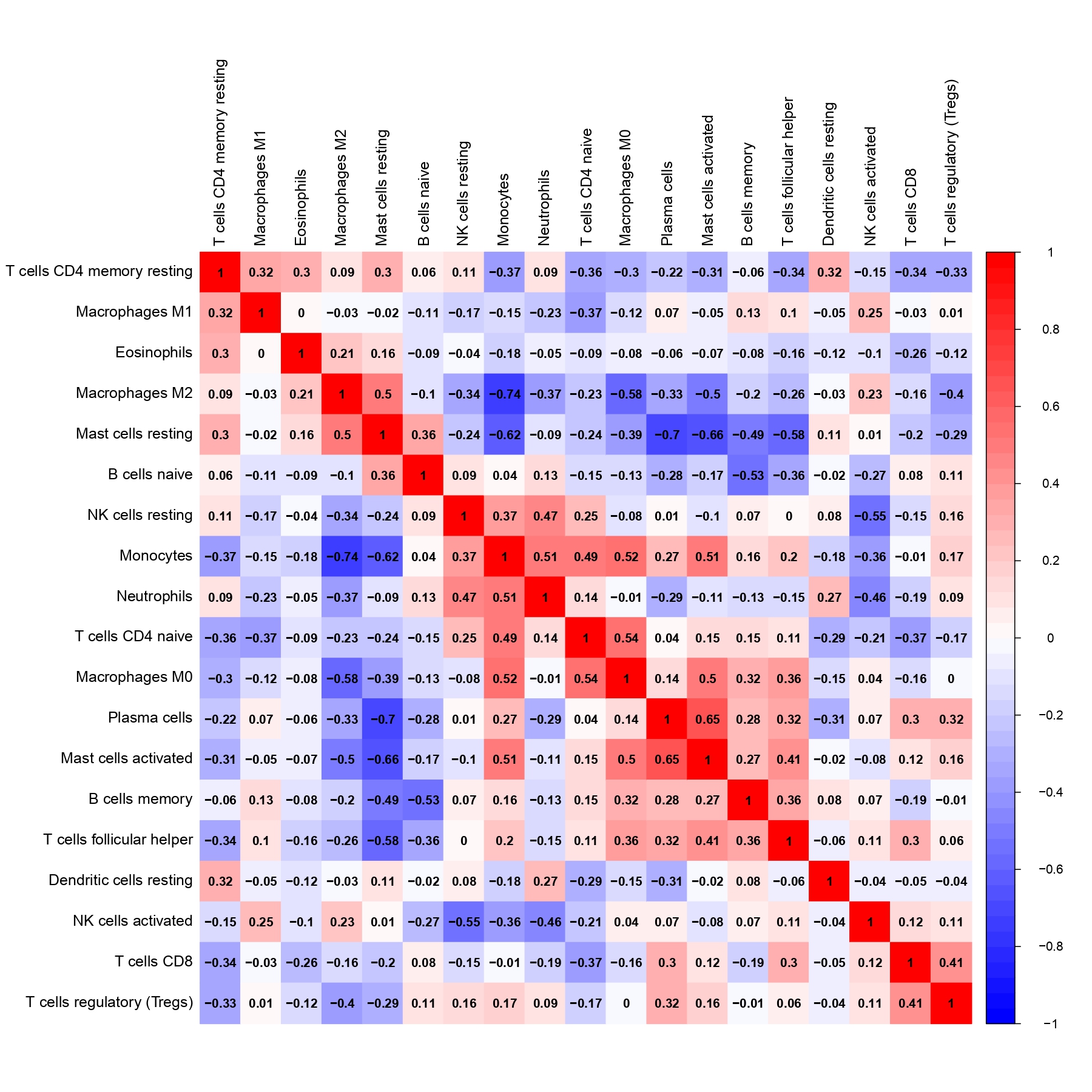

Supplement: Supplementary file 1 [file DataSheet1.ZIP › Supplementary Material Presentation/figure5/figure 5B.jpg]

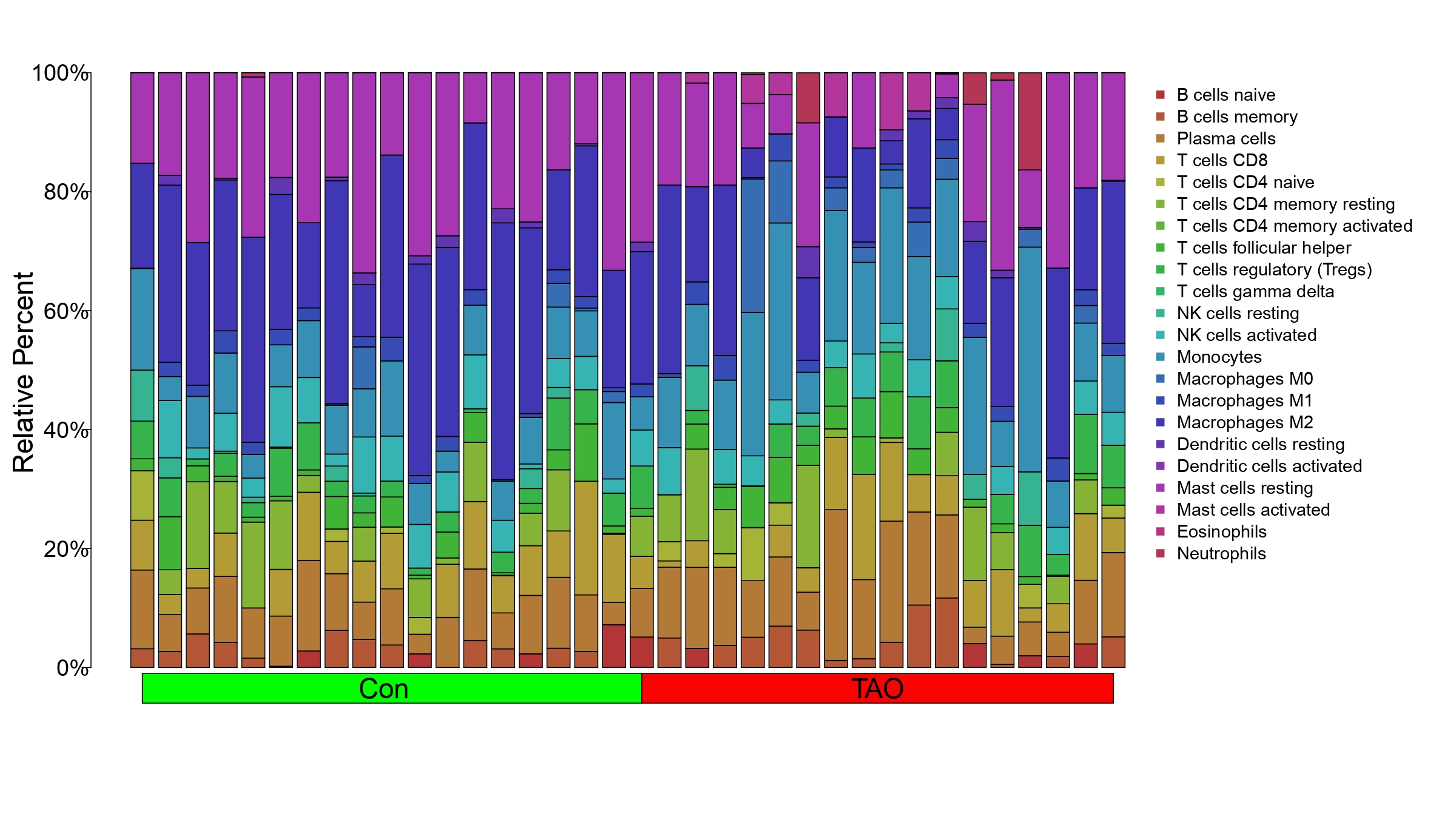

Supplement: Supplementary file 1 [file DataSheet1.ZIP › Supplementary Material Presentation/figure5/figure 5A.jpg]

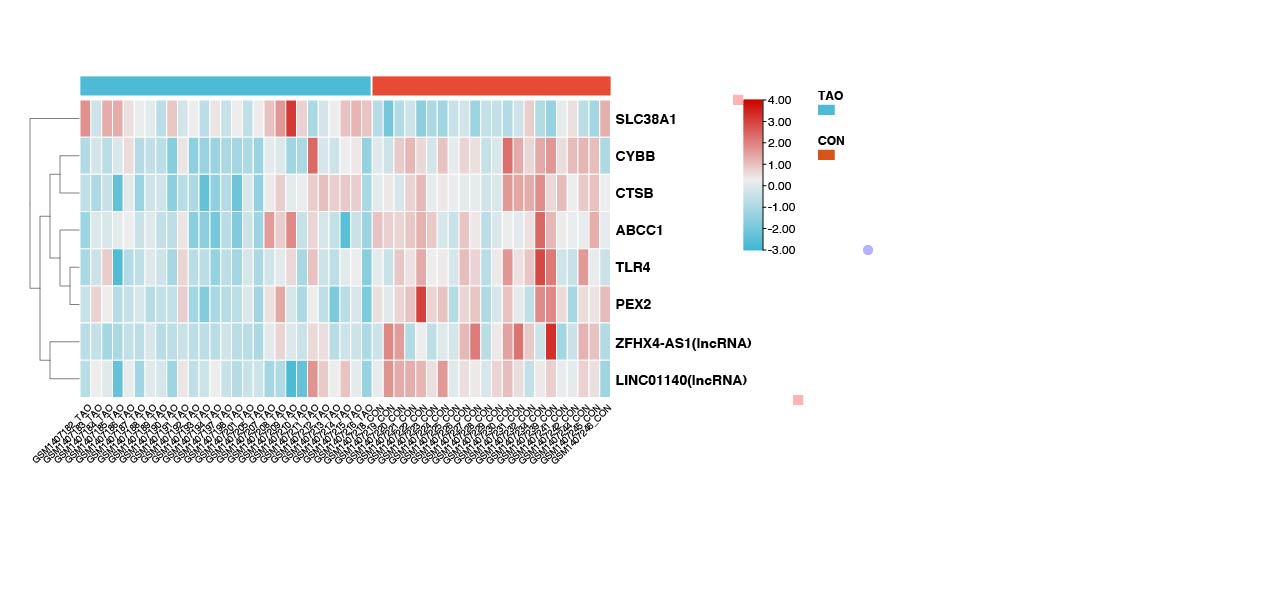

Supplement: Supplementary file 1 [file DataSheet1.ZIP › Supplementary Material Presentation/figure2/figure 2B.jpg]

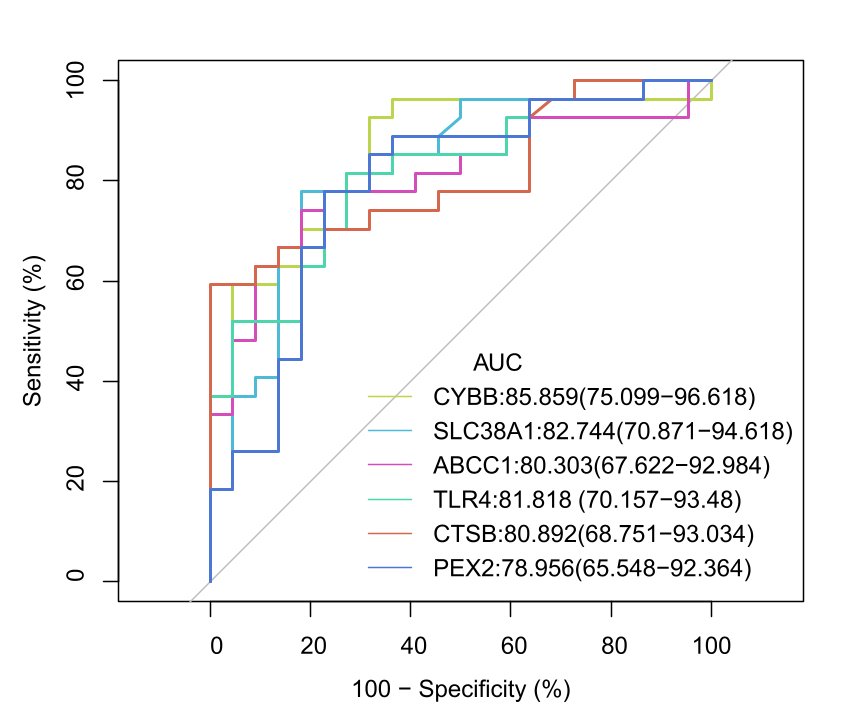

Supplement: Supplementary file 1 [file DataSheet1.ZIP › Supplementary Material Presentation/figure2/figure 2C.jpg]

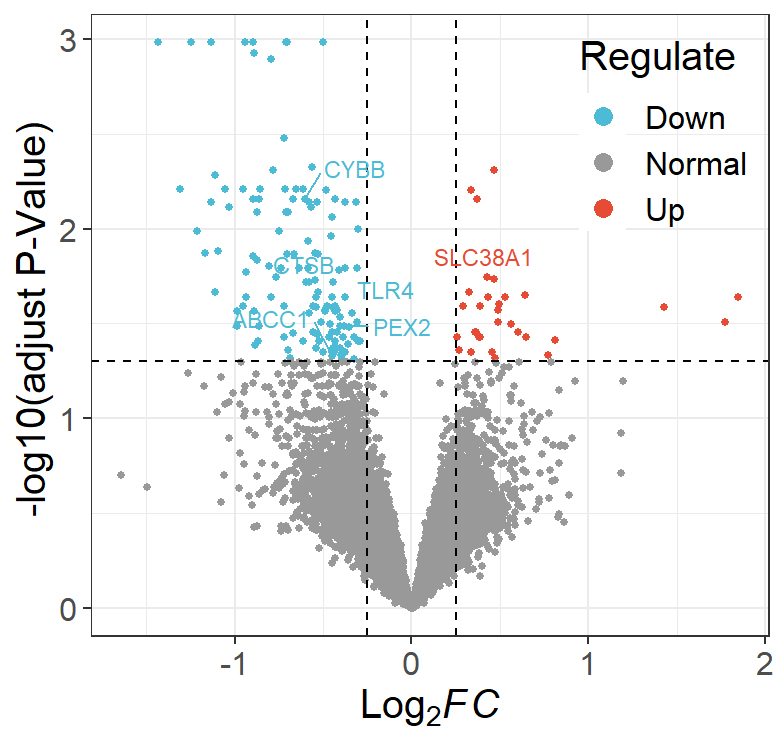

Supplement: Supplementary file 1 [file DataSheet1.ZIP › Supplementary Material Presentation/figure2/figure 2A.jpg]

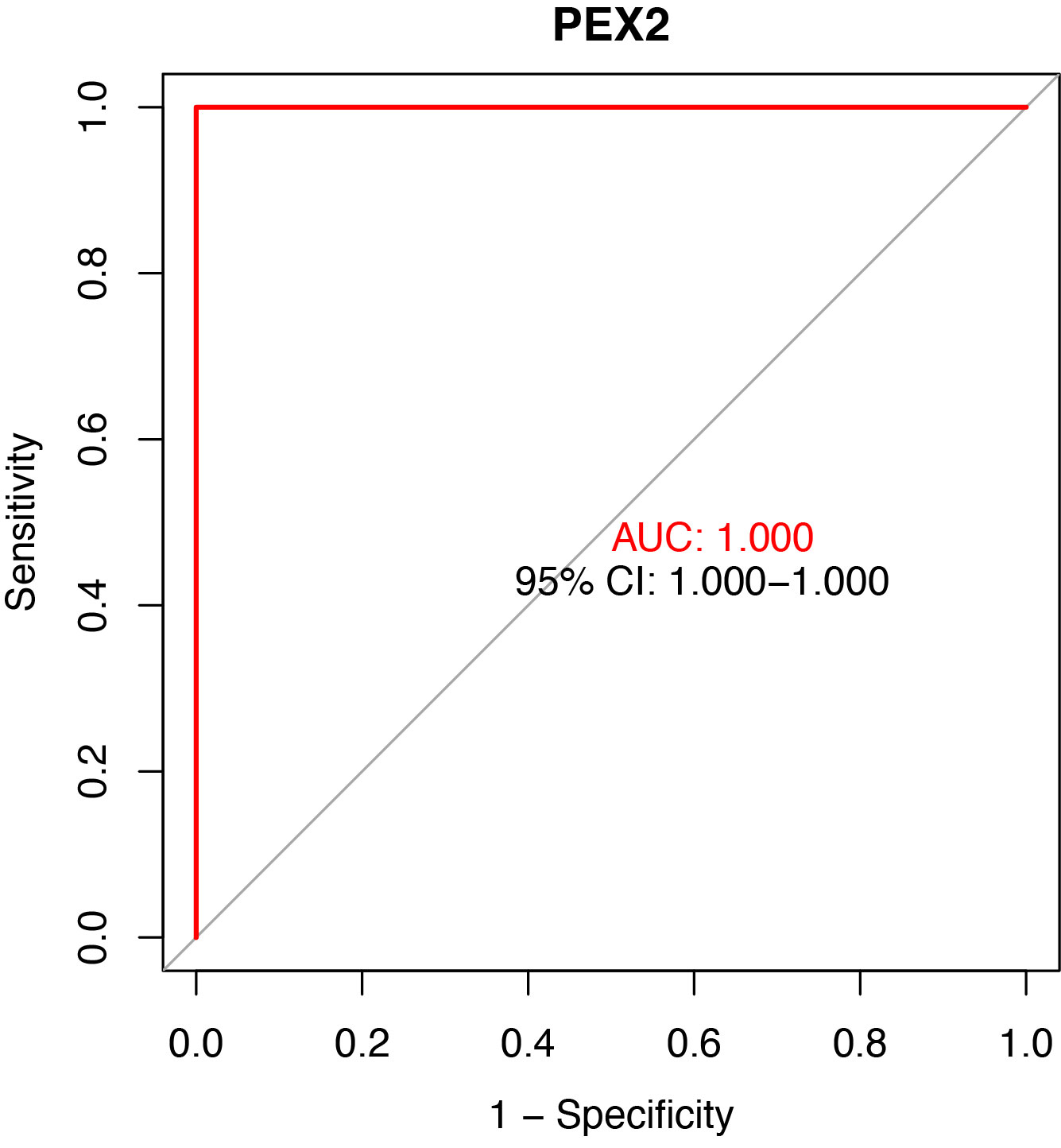

Supplement: Supplementary file 1 [file DataSheet1.ZIP › Supplementary Material Presentation/figure3/figure3E.jpg]

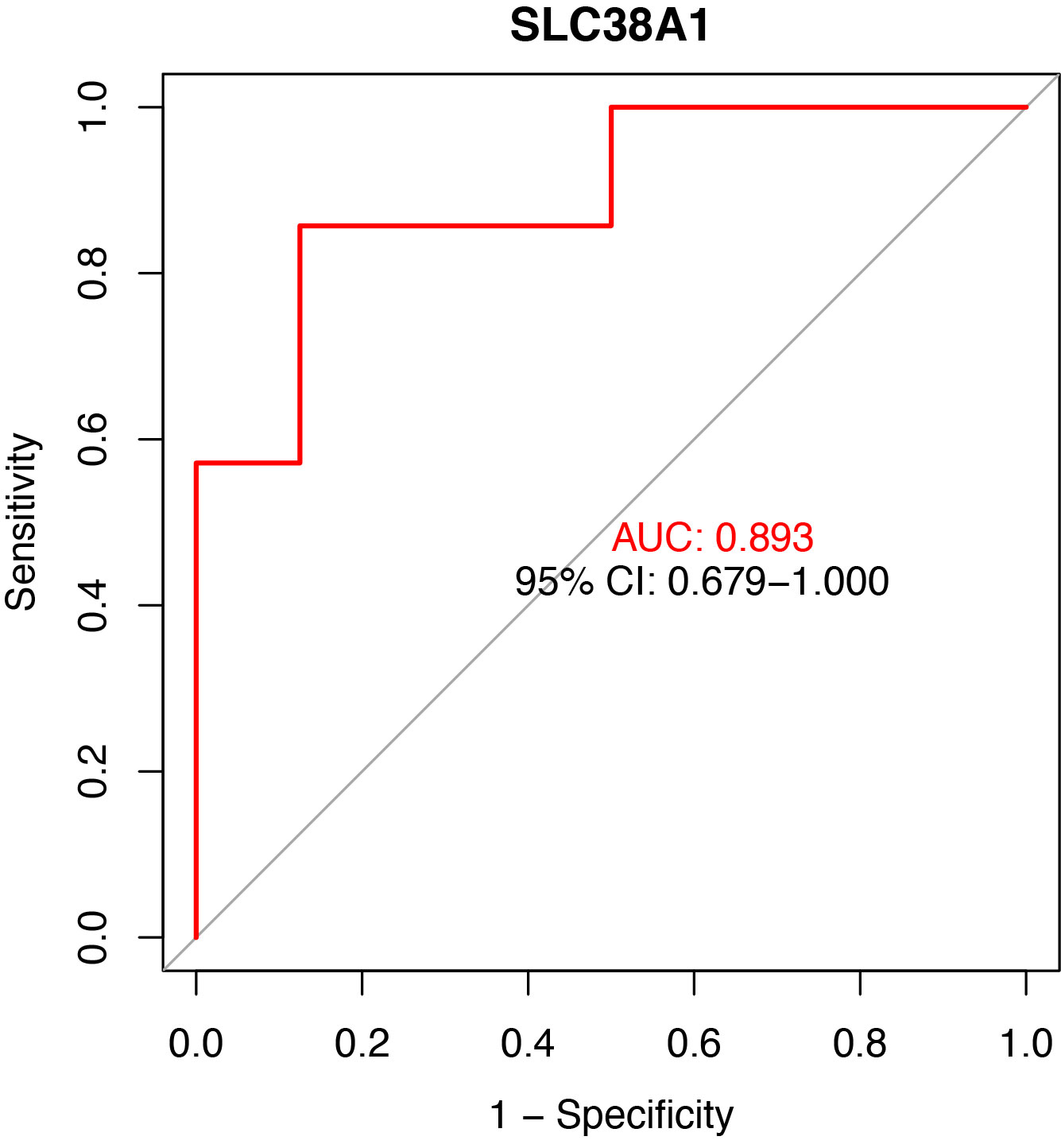

Supplement: Supplementary file 1 [file DataSheet1.ZIP › Supplementary Material Presentation/figure3/figure3D.jpg]

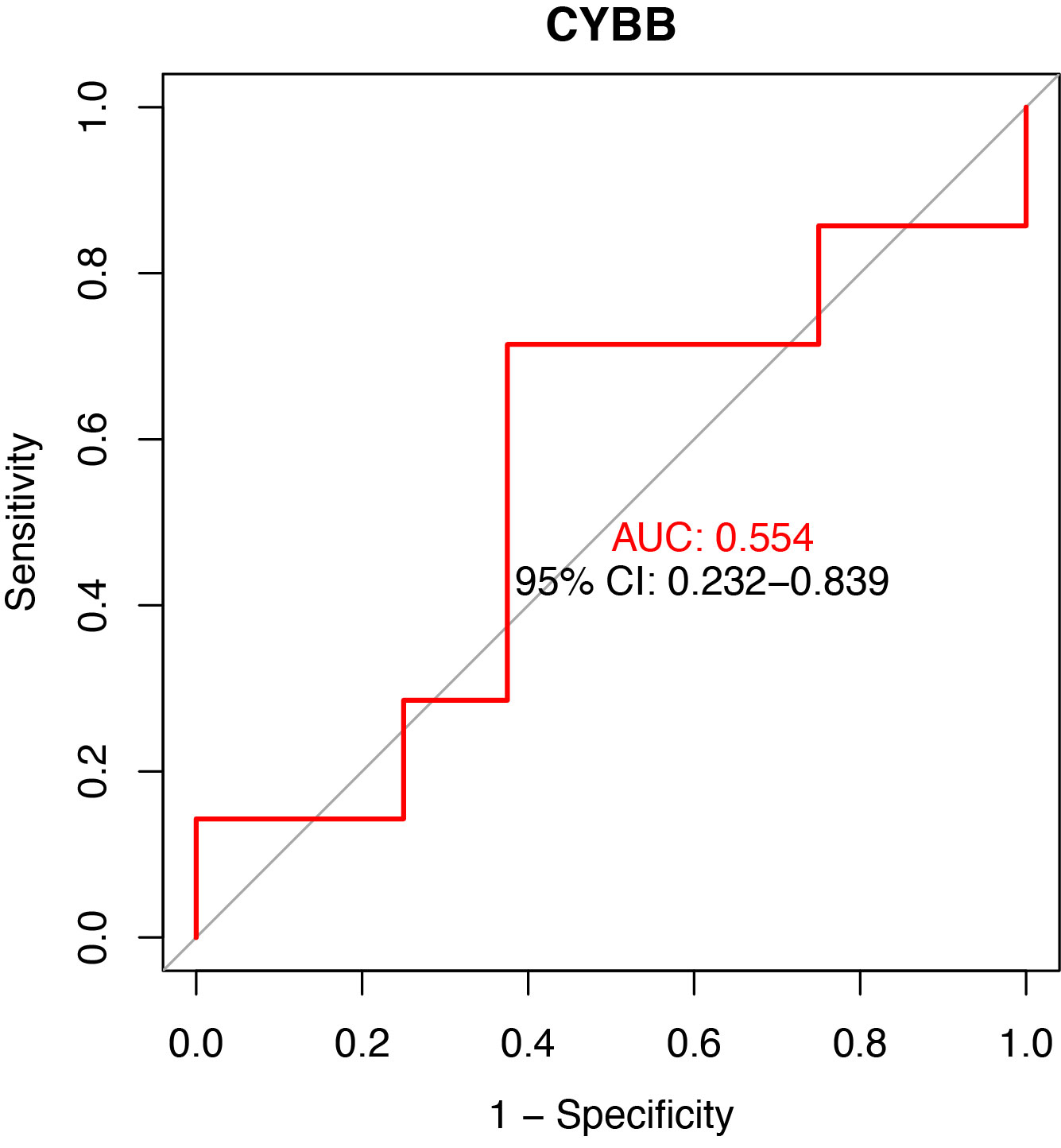

Supplement: Supplementary file 1 [file DataSheet1.ZIP › Supplementary Material Presentation/figure3/figure3F.jpg]

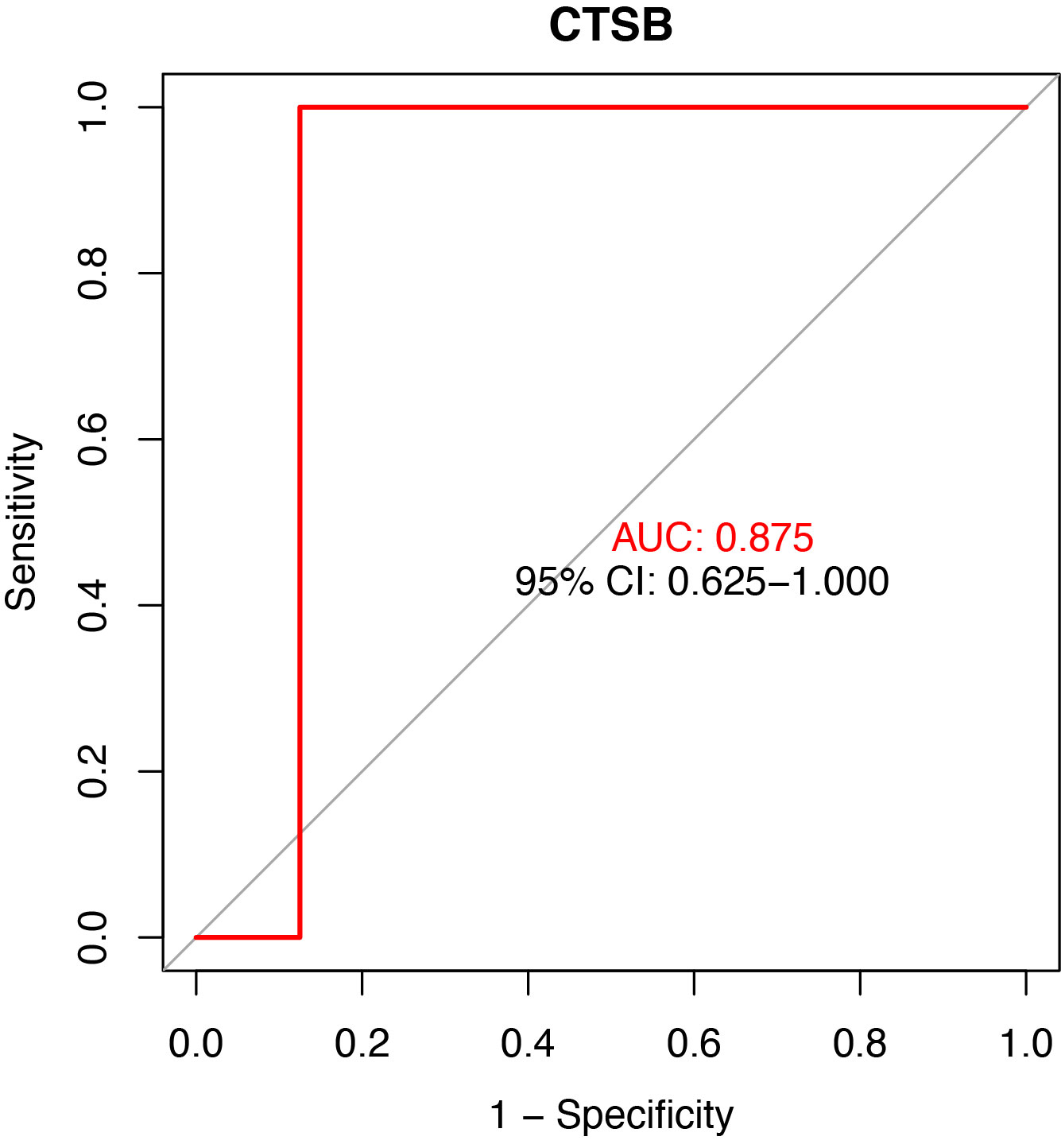

Supplement: Supplementary file 1 [file DataSheet1.ZIP › Supplementary Material Presentation/figure3/figure3C.jpg]

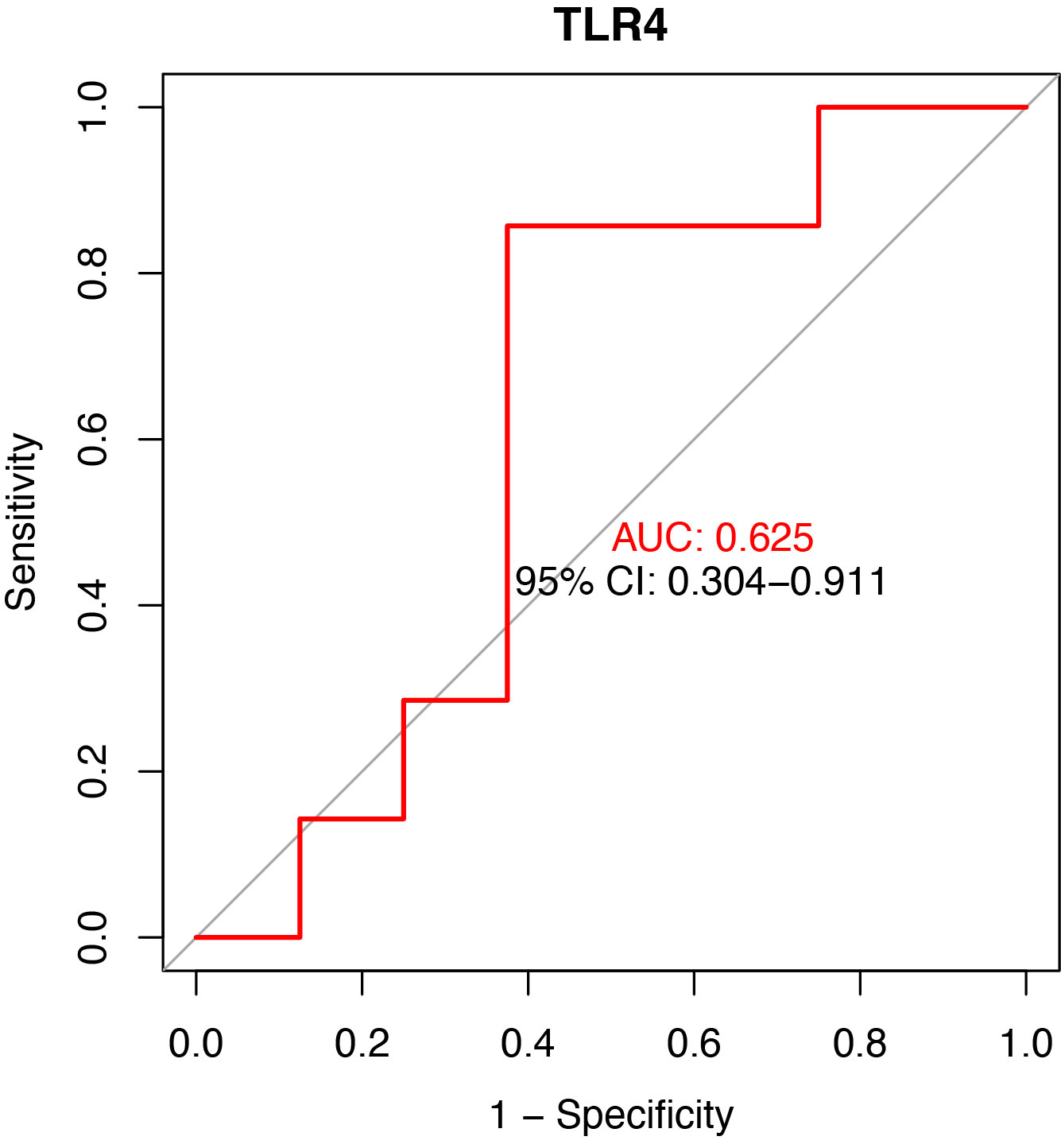

Supplement: Supplementary file 1 [file DataSheet1.ZIP › Supplementary Material Presentation/figure3/figure3B.jpg]

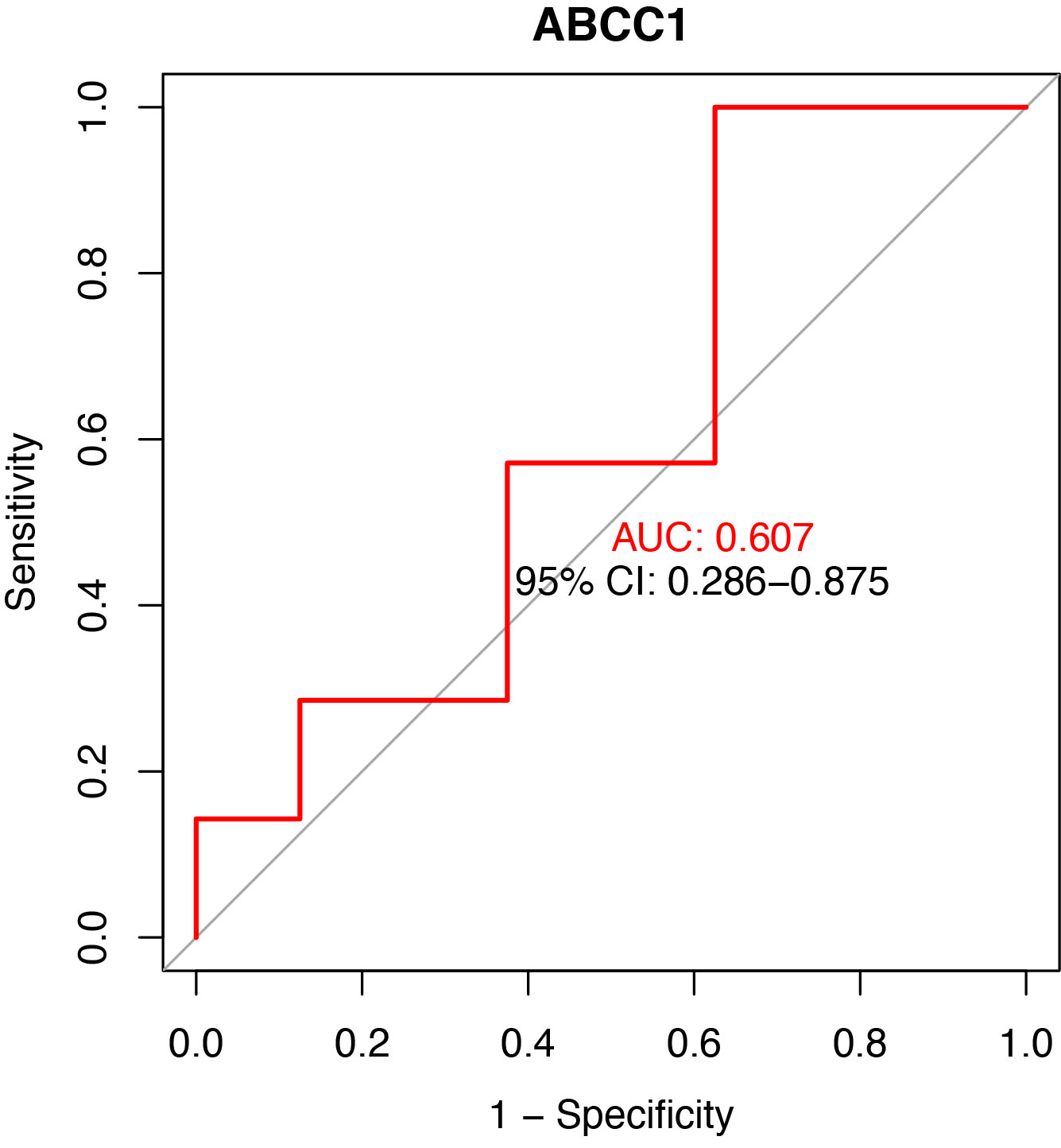

Supplement: Supplementary file 1 [file DataSheet1.ZIP › Supplementary Material Presentation/figure3/figure3A.jpg]

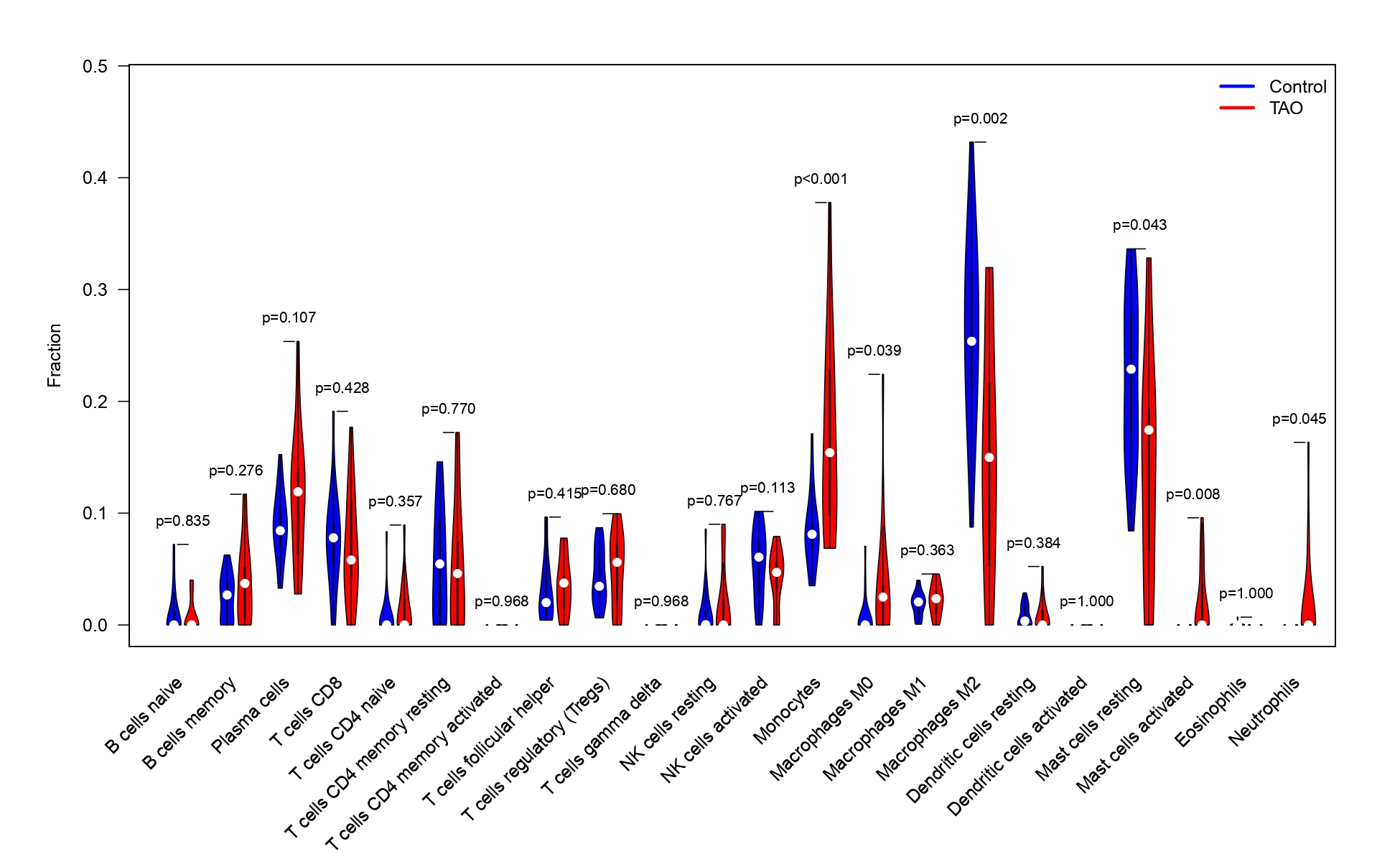

Supplement: Supplementary file 1 [file DataSheet1.ZIP › Supplementary Material Presentation/figure4/figure 4A.jpg]

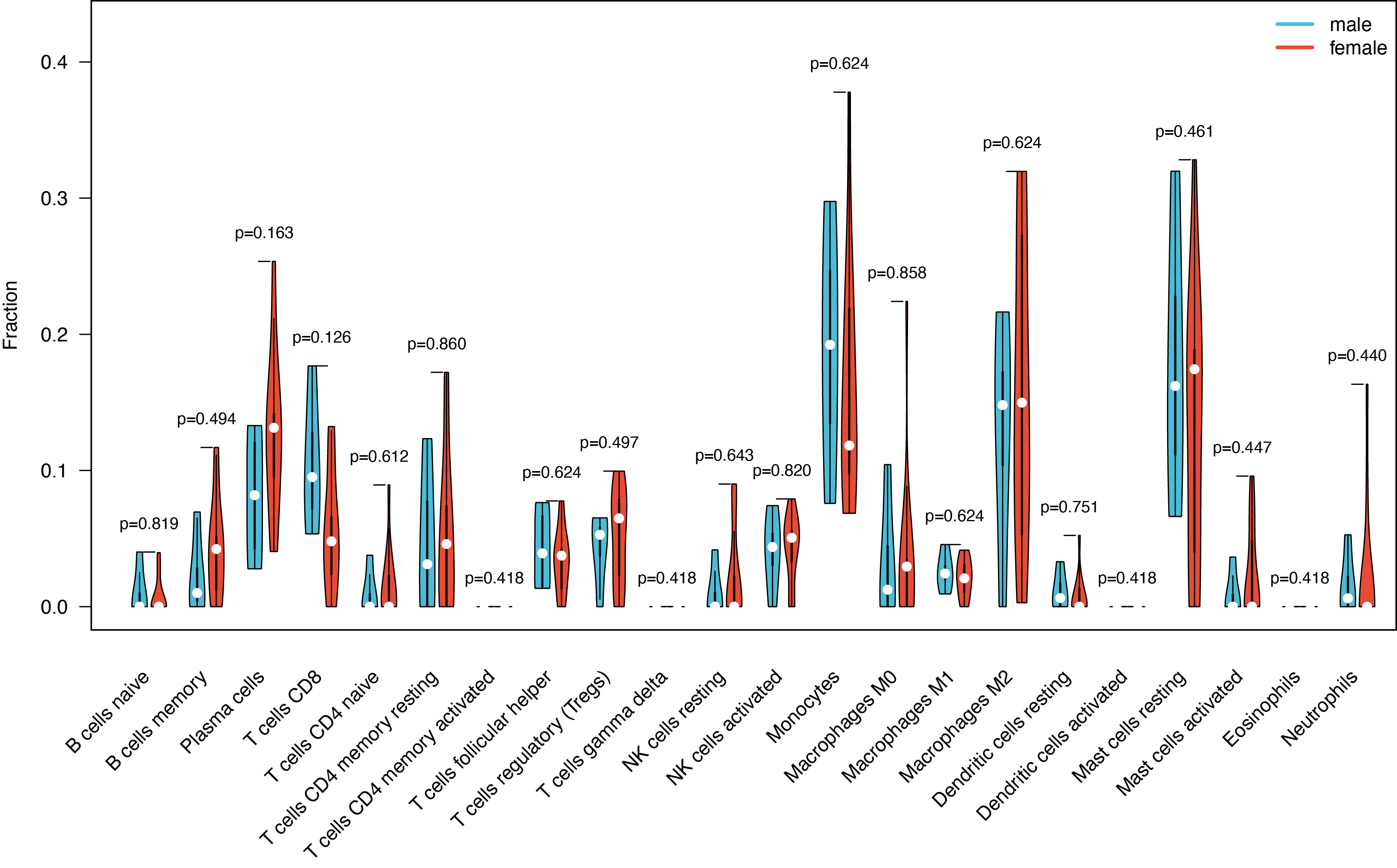

Supplement: Supplementary file 1 [file DataSheet1.ZIP › Supplementary Material Presentation/figure4/figure 4B.jpg]

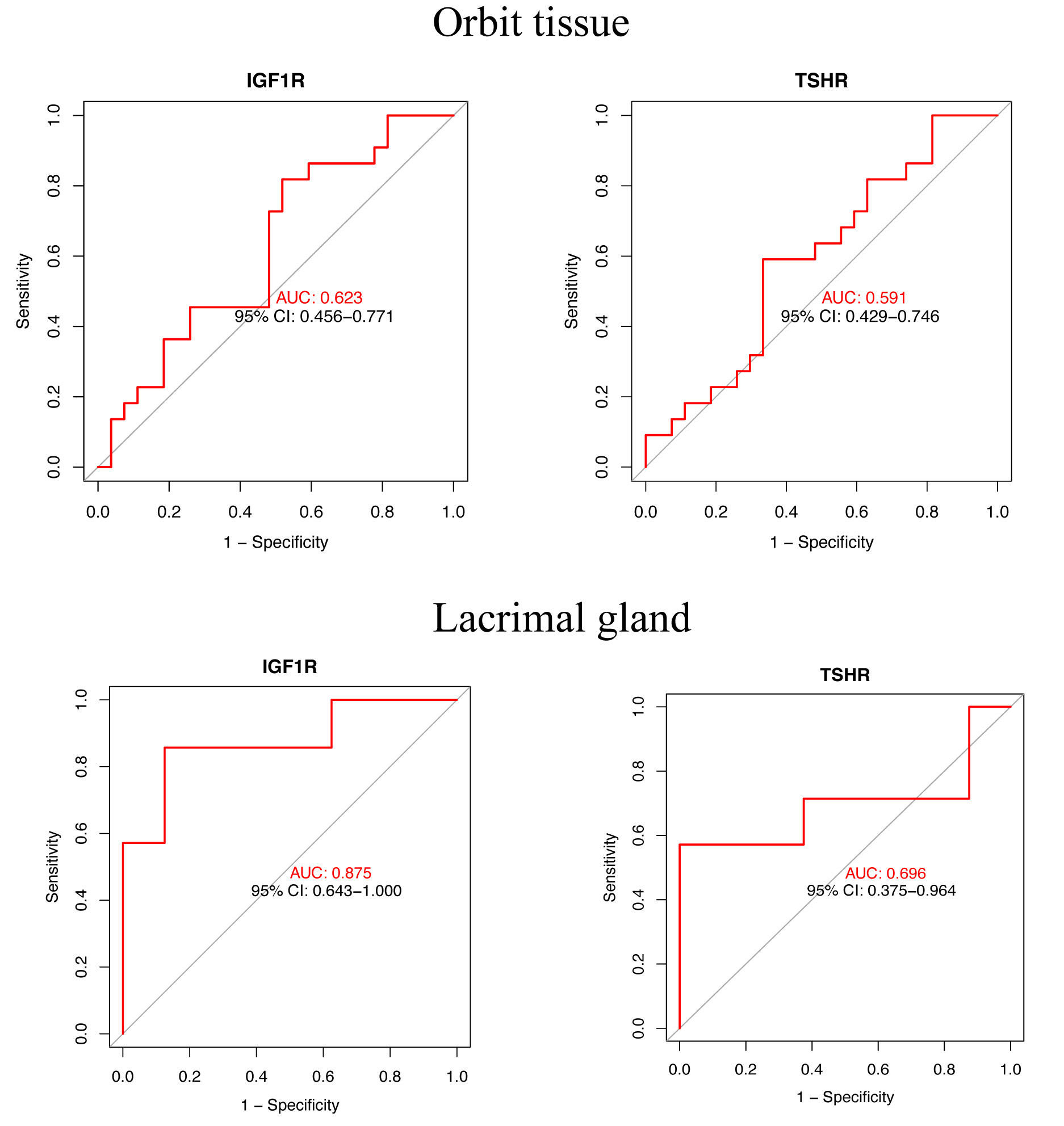

Supplement: Supplementary file 1 [file DataSheet1.ZIP › Supplementary Material Presentation/Supplementary Figure/Supplementary Figure 1.jpg]

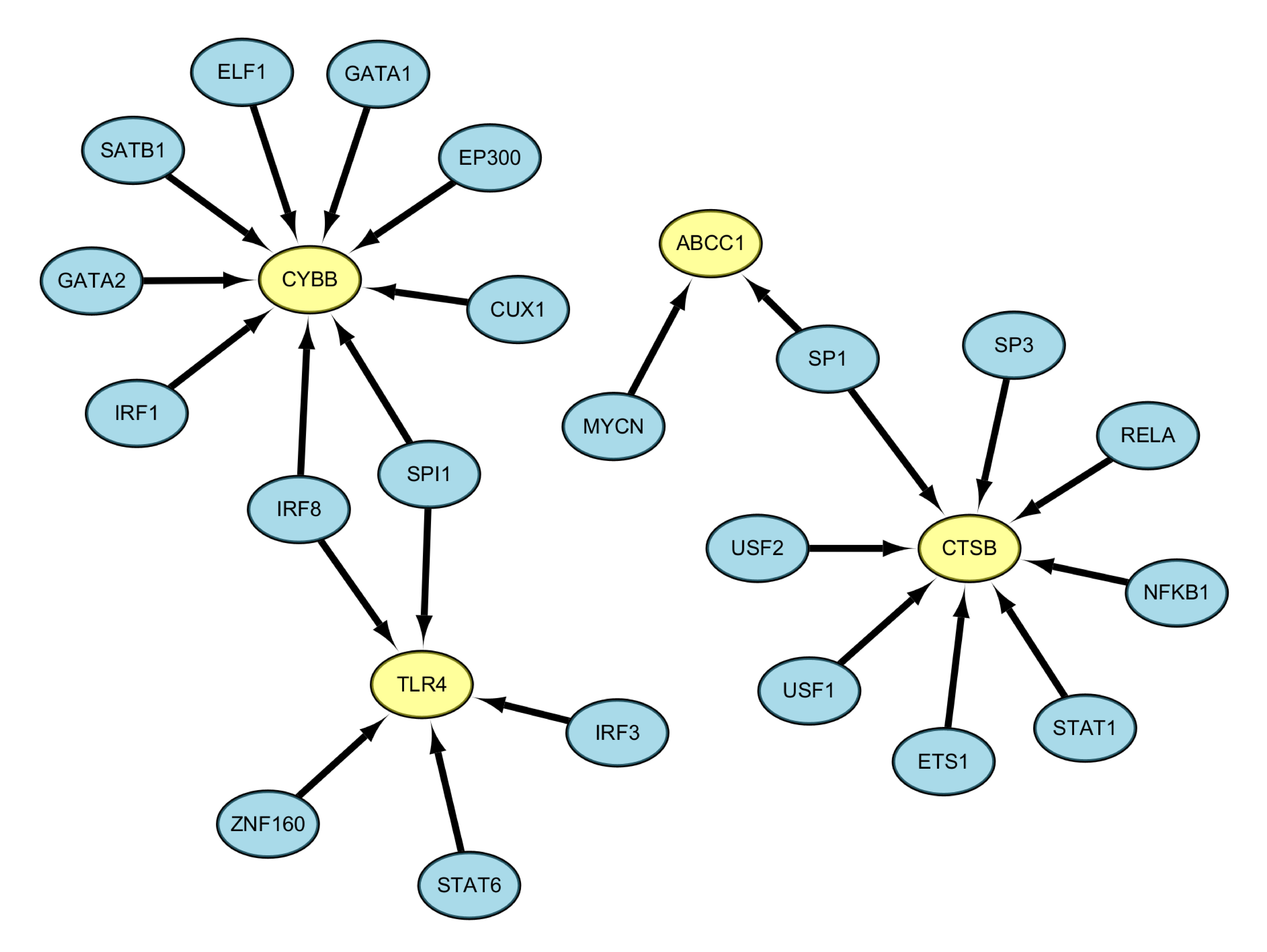

Supplement: Supplementary file 1 [file DataSheet1.ZIP › Supplementary Material Presentation/figure8/figure 8B.jpg]

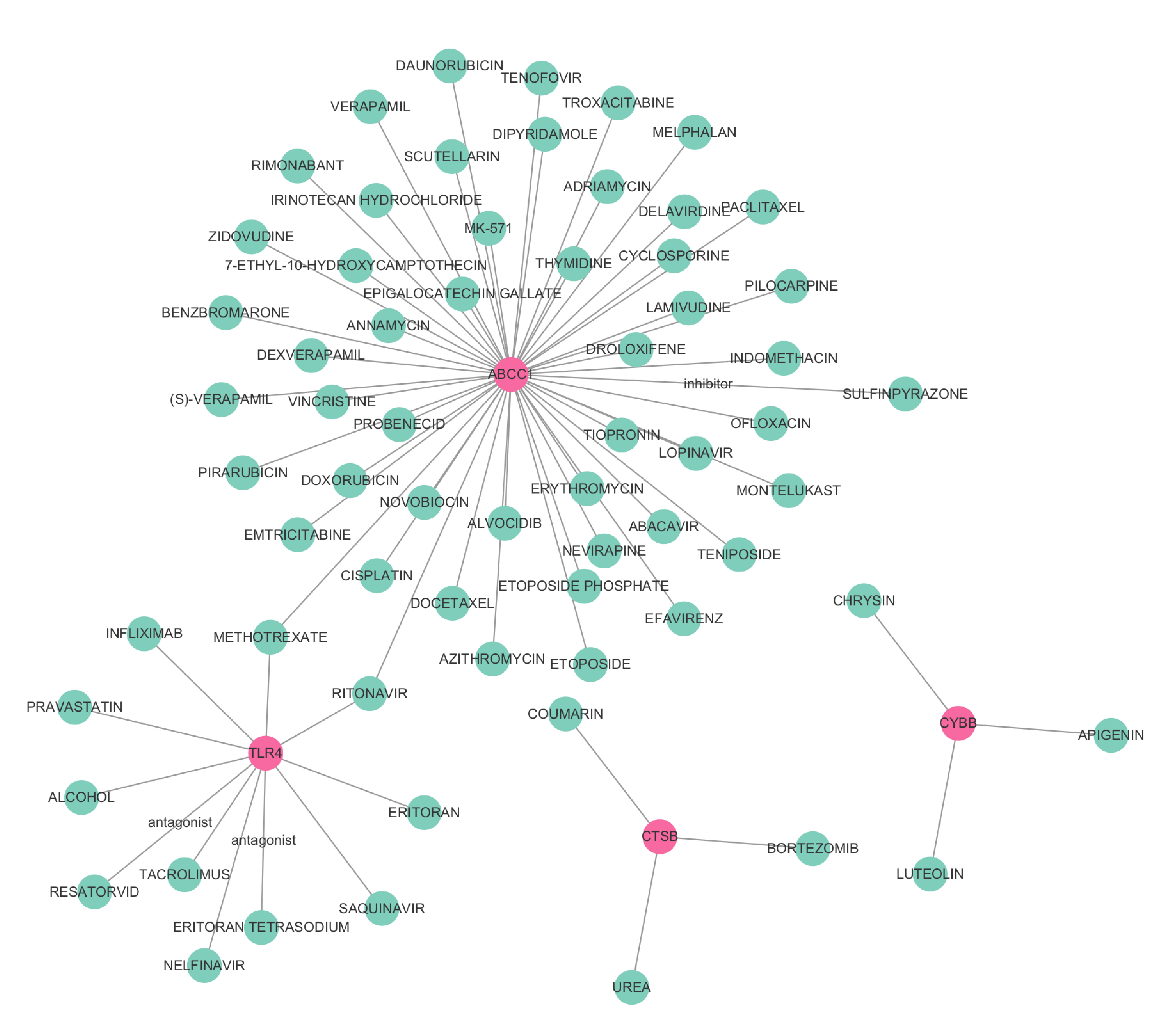

Supplement: Supplementary file 1 [file DataSheet1.ZIP › Supplementary Material Presentation/figure8/figure 8A.jpg]

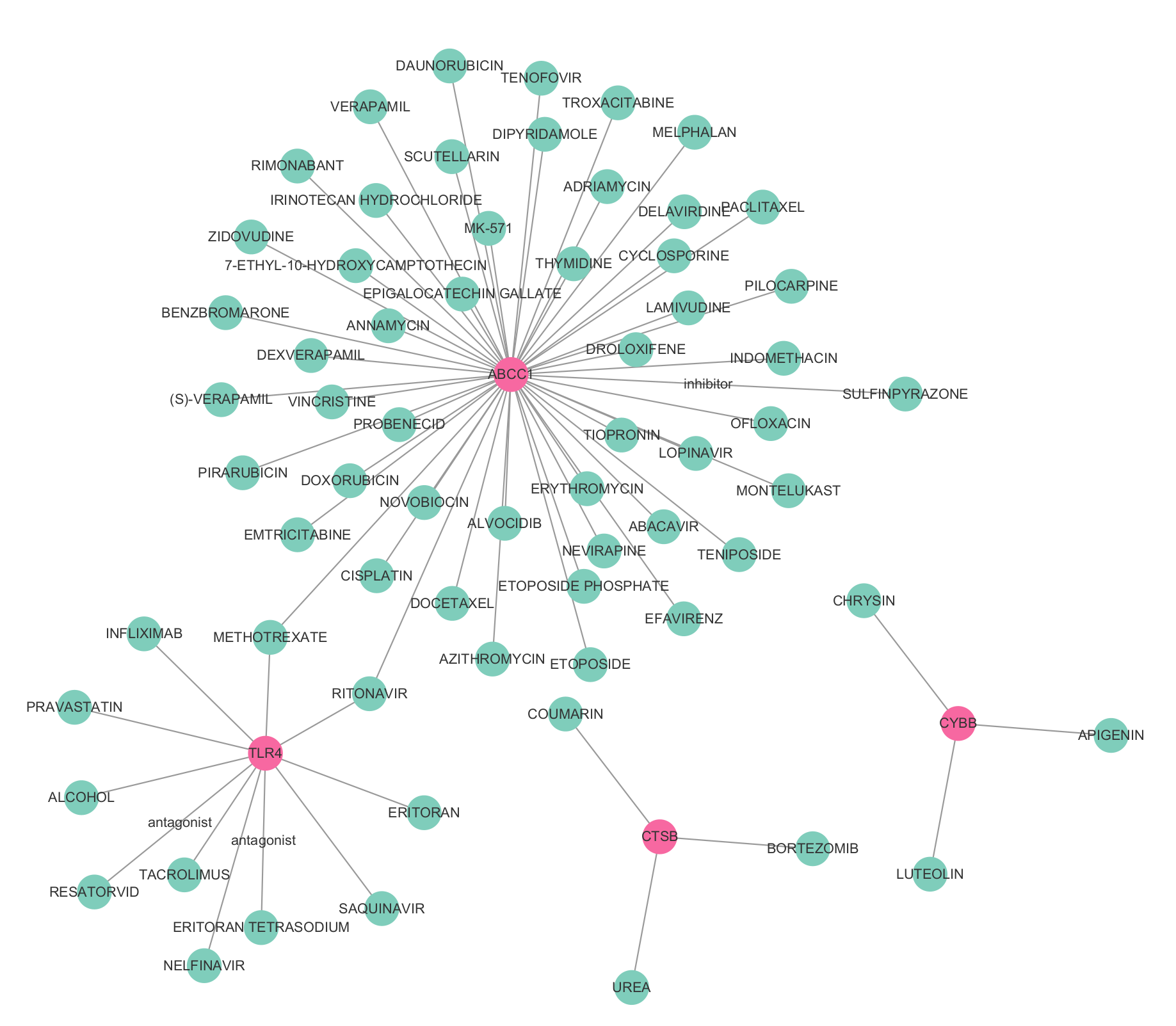

Supplement: Supplementary file 1 [file DataSheet1.ZIP › Supplementary Material Presentation/figure8/figure 8A.png]

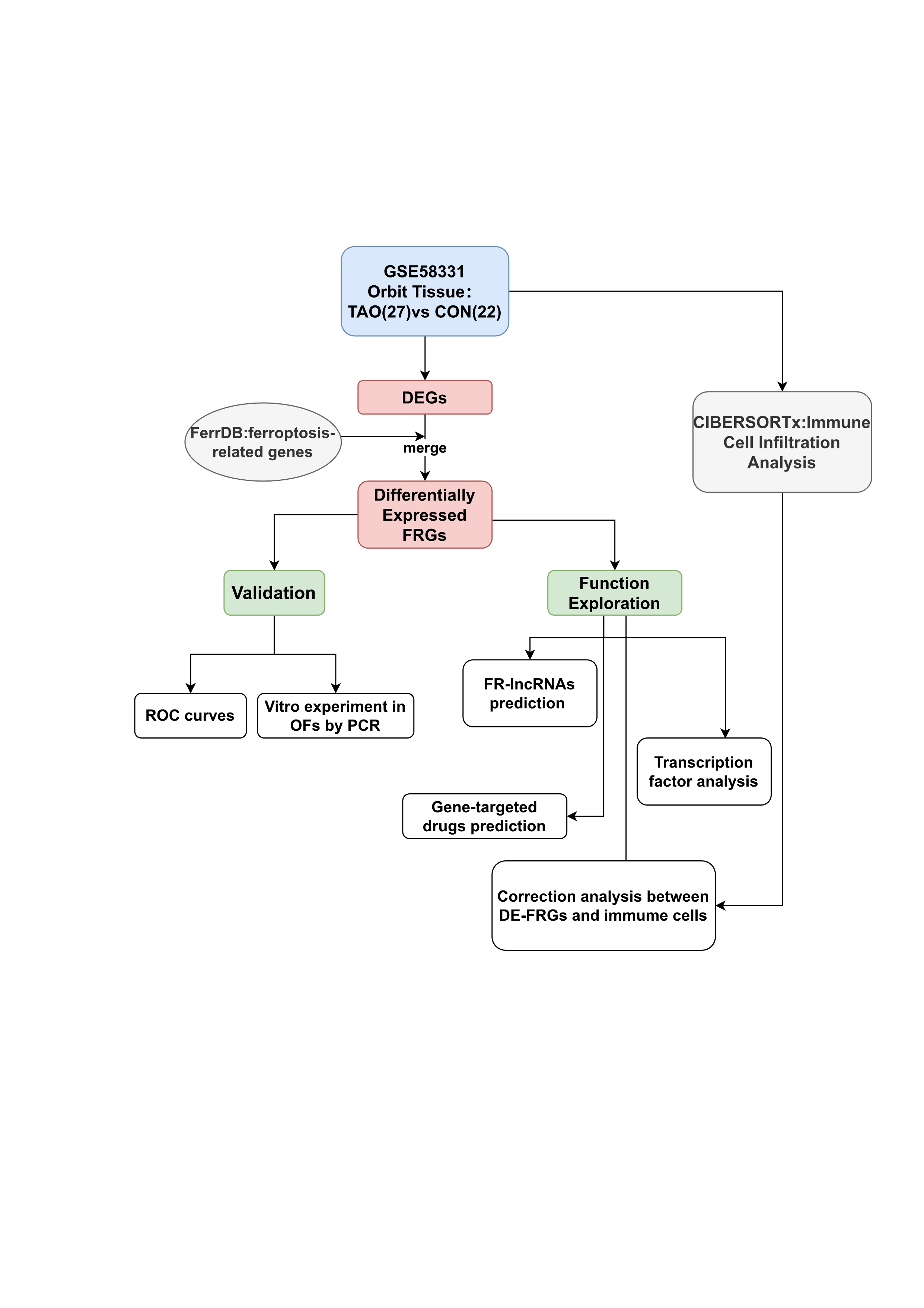

Supplement: Supplementary file 1 [file DataSheet1.ZIP › Supplementary Material Presentation/figure1/figure1.jpg]

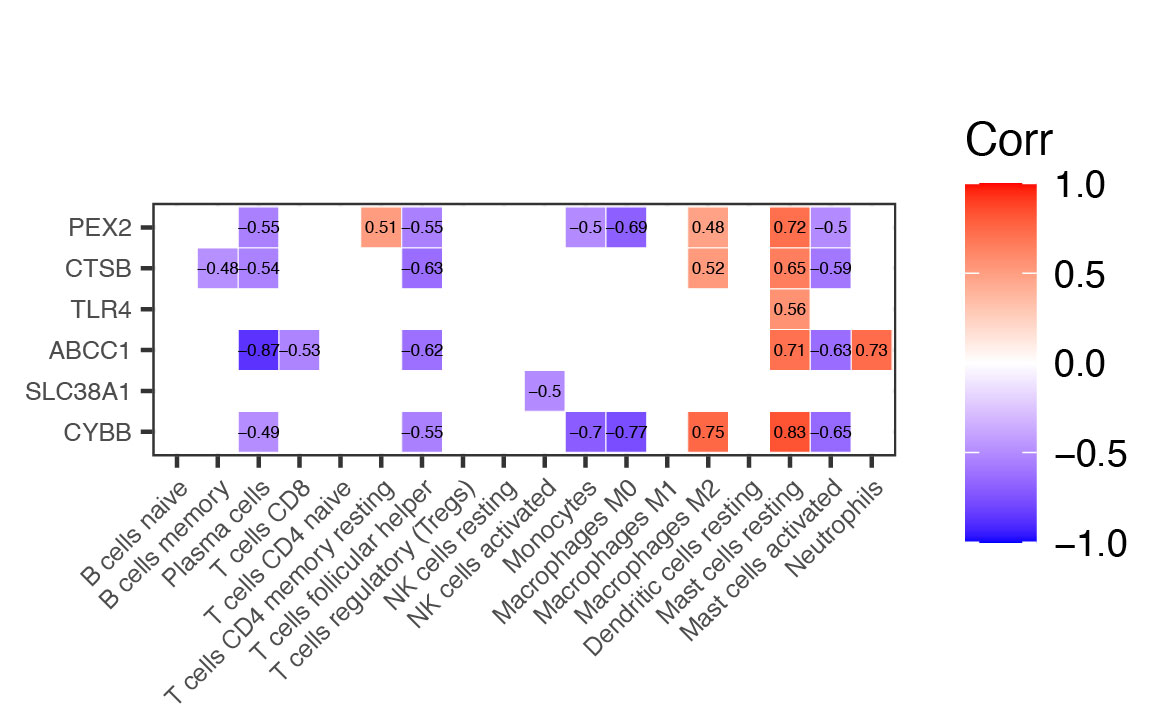

Supplement: Supplementary file 1 [file DataSheet1.ZIP › Supplementary Material Presentation/figure6/figure 6.jpg]

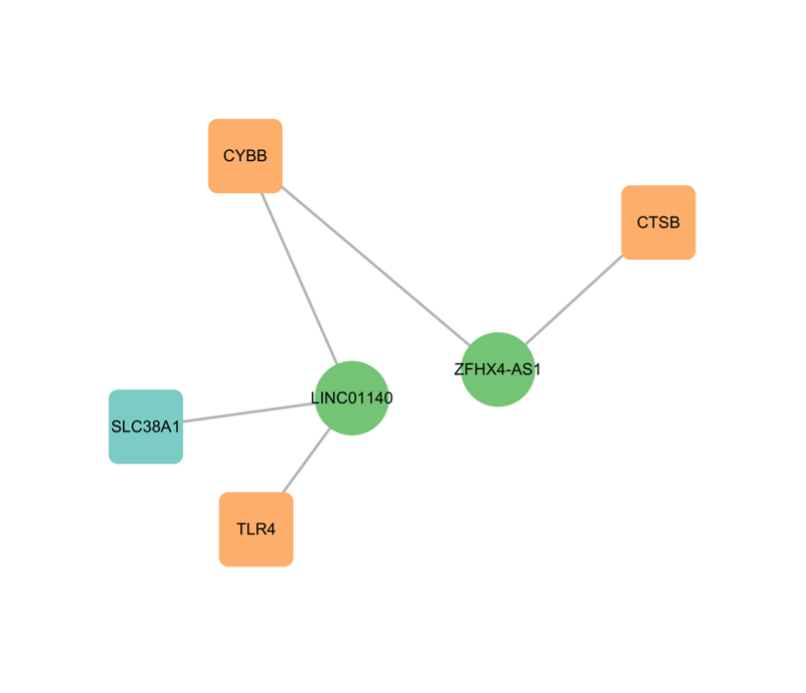

Supplement: Supplementary file 1 [file DataSheet1.ZIP › Supplementary Material Presentation/figure7/figure 7.jpg]

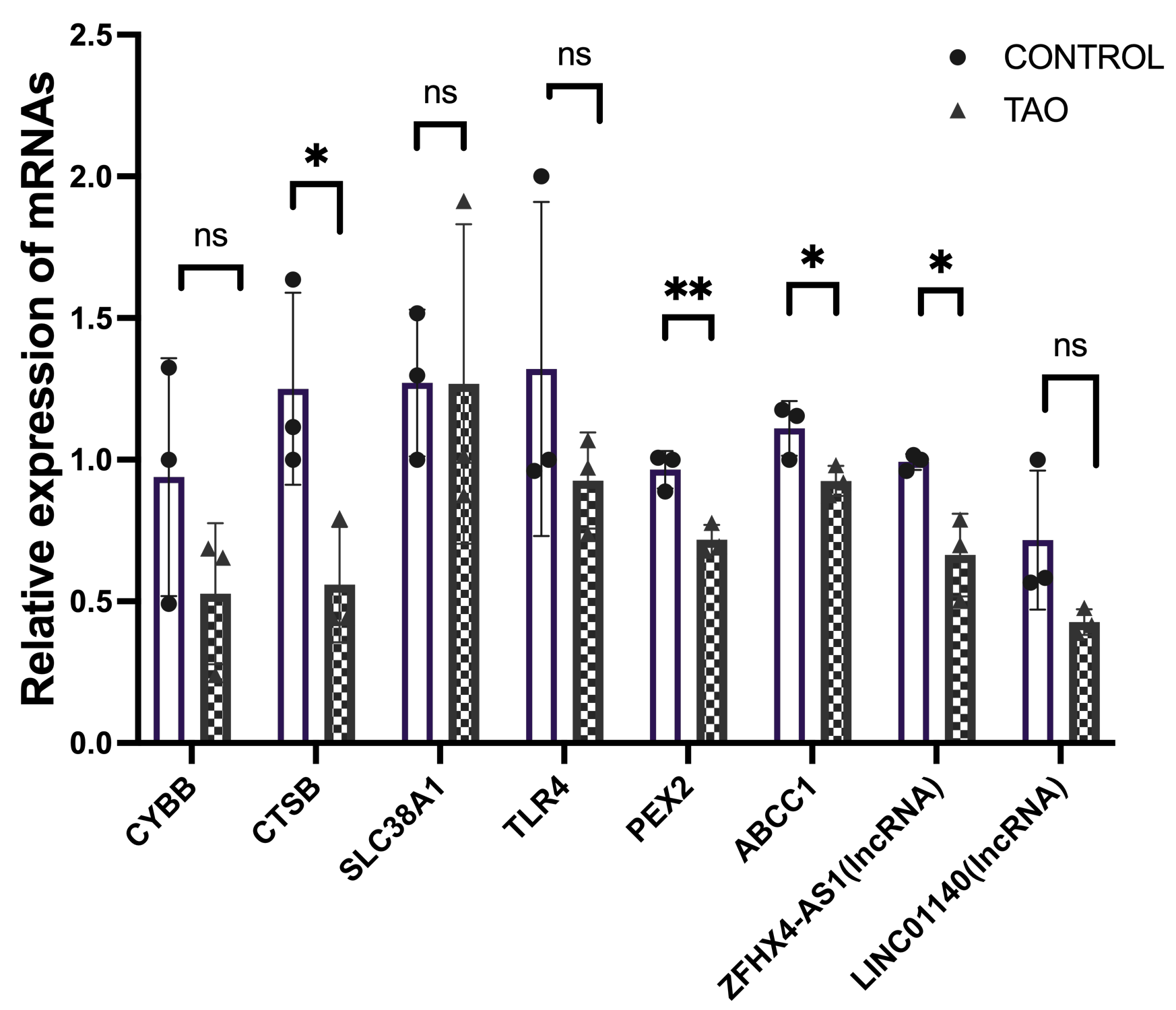

Supplement: Supplementary file 1 [file DataSheet1.ZIP › Supplementary Material Presentation/figure9/figure9.tiff]
